# Functional characterization of helminth-associated Clostridiales reveals covariates of Treg differentiation

**DOI:** 10.1186/s40168-024-01793-1

**Published:** 2024-05-10

**Authors:** Shushan Sargsian, Octavio Mondragón-Palomino, Alannah Lejeune, Defne Ercelen, Wen-Bing Jin, Alan Varghese, Yvonne A. L. Lim, Chun-Jun Guo, P’ng Loke, Ken Cadwell

**Affiliations:** 1 Department of Microbiology, New York University Grossman School of Medicine, New York, NY 10016 USA; 2 Laboratory of Parasitic Diseases, National Institute of Allergy and Infectious Diseases, National Institutes of Health, Bethesda, MD 20892 USA; 3 Department of Medicine, Division of Gastroenterology and Hepatology, New York University Langone Health, New York, NY 10016 USA; 4 Weill Cornell Medicine, Jill Roberts Institute for Research in Inflammatory Bowel Disease, Cornell University, New York, NY 10021 USA; 5 Department of Cell Biology, New York University Grossman School of Medicine, New York, NY 10016 USA; 6 Department of Parasitology, Faculty of Medicine, Universiti Malaya, Kuala Lumpur, 50603 Malaysia; 7 Department of Medicine, Division of Gastroenterology and Hepatology, University of Pennsylvania Perelman School of Medicine, Philadelphia, PA 19104 USA; 8 Department of Systems Pharmacology and Translational Therapeutics, University of Pennsylvania Perelman School of Medicine, Philadelphia, PA 19104 USA; 9 Department of Pathology and Laboratory Medicine, University of Pennsylvania Perelman School of Medicine, Philadelphia, PA 19104 USA

**Keywords:** Clostridia, Helminth, *Trichuris*, Microbiome, Immune modulation, Regulatory T cells, Metabolites, Bacterial enzymes

## Abstract

**Background:**

Parasitic helminths influence the composition of the gut microbiome. However, the microbiomes of individuals living in helminth-endemic regions are understudied. The Orang Asli, an indigenous population in Malaysia with high burdens of the helminth *Trichuris trichiura*, display microbiotas enriched in Clostridiales, an order of spore-forming obligate anaerobes with immunogenic properties. We previously isolated novel Clostridiales that were enriched in these individuals and found that a subset promoted the *Trichuris* life cycle. In this study, we aimed to further characterize the functional properties of these bacteria.

**Results:**

Clostridiales isolates were profiled for their ability to perform 57 enzymatic reactions and produce short-chain fatty acids (SCFAs) and hydrogen sulfide, revealing that these bacteria were capable of a range of activities associated with metabolism and host response. Consistent with this finding, monocolonization of mice with individual isolates identified bacteria that were potent inducers of regulatory T-cell (Treg) differentiation in the colon. Comparisons between variables revealed by these studies identified enzymatic properties correlated with Treg induction and *Trichuris* egg hatching.

**Conclusion:**

We identified Clostridiales species that are sufficient to induce high levels of Tregs. We also identified a set of metabolic activities linked with Treg differentiation and *Trichuris* egg hatching mediated by these newly isolated bacteria. Altogether, this study provides functional insights into the microbiotas of individuals residing in a helminth-endemic region.

Video Abstract

**Supplementary Information:**

The online version contains supplementary material available at 10.1186/s40168-024-01793-1.

## Introduction

The gut microbiota and its enzymatic byproducts impact numerous aspects of host physiology including metabolism and immunity [1–9]. The composition of the microbiota is associated with numerous disease conditions and heavily influenced by geography and lifestyle [10–18]. However, microbiome research is dominated by studies examining individuals in highly developed countries [19, 20]. Environmental variables absent in these populations contribute to differences in their microbiome compared with individuals residing in other regions. For instance, parasitic worms known as helminths cohabitate the gastrointestinal tract alongside bacteria and influence the diversity and composition of the microbiota [21–25]. Helminths colonize 1.5 billion people, or roughly 24% of the world population, with the highest prevalence reported in tropical and subtropical areas in Africa, Asia, and South America [26–28]. Examining the properties of intestinal bacteria enriched in individuals from helminth-endemic regions may broaden our understanding of microbiota function.

Helminth infection can cause disease involving malnutrition, impaired growth, dysentery, intestinal obstruction, and anemia [26, 27]. The incidence of helminth infections is also negatively linked to the incidence of immune-mediated disorders on a global scale [29, 30]. The immune response generated against helminths includes the differentiation and expansion of regulatory T cells (Tregs), which suppress the development of autoimmune and inflammatory diseases [31–36]. Immune modulation by helminths can occur either by direct effects on the host immune system or indirectly through the microbiota [37–41]. For example, we and others have shown that expansion of the Clostridiales order during helminth colonization ameliorates disease in mouse models of inflammatory bowel disease and allergic asthma [41, 42]. We also found that Clostridiales were enriched in the gut microbiome of the Orang Asli, an indigenous population in rural Malaysia. Clostridiales were enriched in individuals with high burdens of the whipworm *Trichuris trichiura*, and deworming medication reduced the relative abundance of Clostridiales [42].

Clostridiales and the Clostridia class to which they belong are Gram-positive, spore-forming anaerobes that influence host physiology, such as through promoting differentiation of Tregs [5, 43, 44]. Based on the above association with helminths, we recently used a chloroform-based enrichment protocol to isolate and sequence the genomes of spore-forming bacteria from the feces of helminth-colonized Orang Asli [45]. This approach identified 13 Clostridiales species, most of which represented poorly characterized taxa. Metagenomics analysis of a large number of Malaysians confirmed the association of these Clostridiales members with the Orang Asli population and identified a specific association between the Peptostreptococcaceae family and helminth colonization. Also, we found that Peptostreptococcaceae isolates were strong inducers of *Trichuris* egg hatching, suggesting that Clostridiales enriched during helminth colonization contain properties that support the helminth life cycle. However, the functional properties of these bacteria remain unknown. Here, we examined the enzymatic and metabolomic activity of isolated bacteria and their ability to induce Treg differentiation and identified specific enzymatic properties that correlated with Treg induction and *Trichuris* egg hatching.

## Results

### Study design

**Fig. 1:**
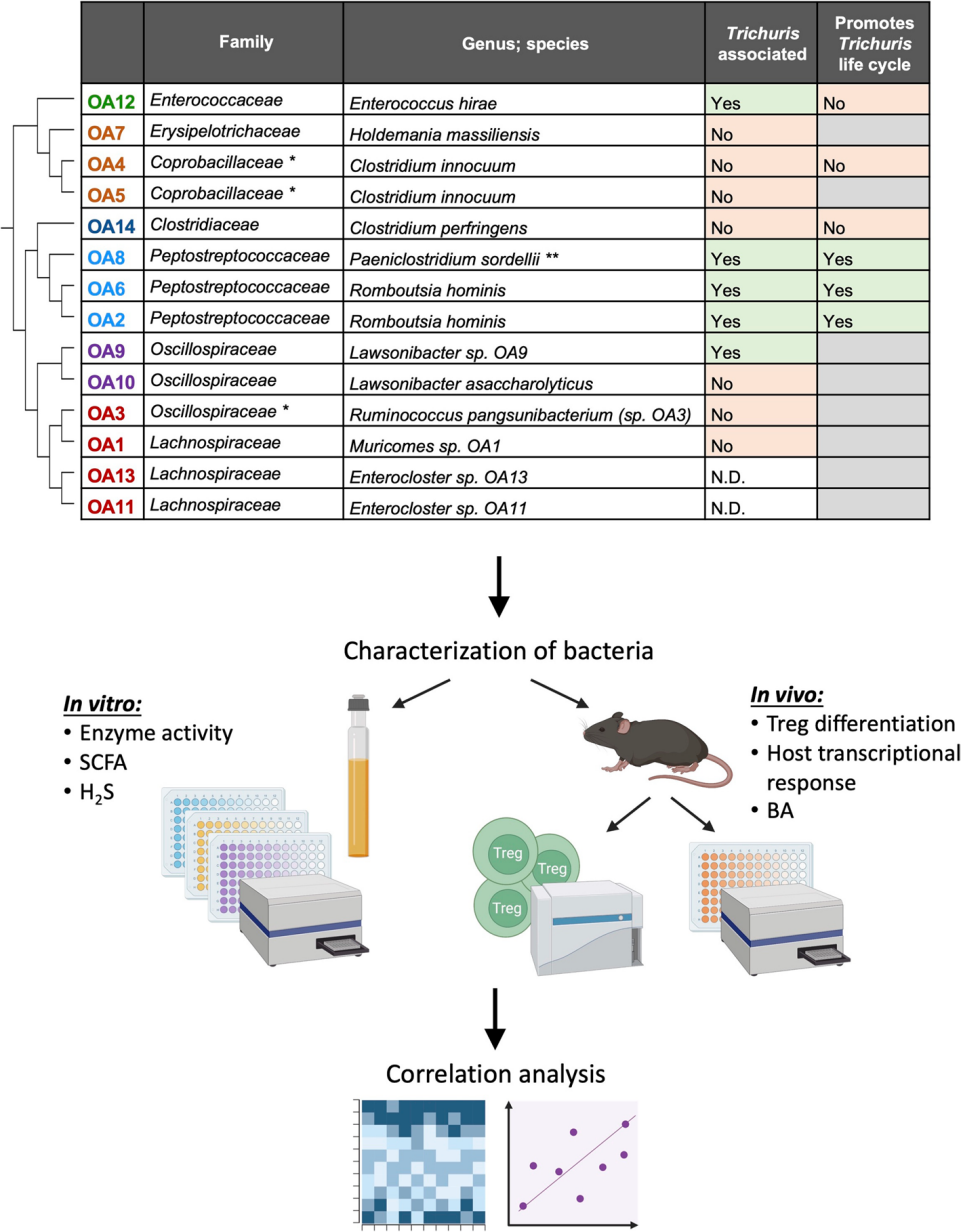
Study design for characterization of the OA isolates. List of OA isolates with updated taxonomic classifications (top) and schematic summarizing the study design (bottom). The phylogenetic tree to the left of the table depicts the relationships between OA isolates. The column labeled “*Trichuris* associated” refers to whether the abundance of the isolate decreased in microbiomes after deworming treatment, with “N.D.” denoting isolates that were not detected [45]. The column labeled “Promotes *Trichuris* life cycle” refers to whether the isolate promotes *Trichuris muris* egg hatching, with gray cells denoting isolates that were not tested [45]. *OA4 and OA5 were classified as members of the Erysipelotrichaceae family, and OA3 was classified as a member of the Lachnospiraceae family at the time of our previous publication [45]. **OA8 was classified as *Paraclostridium sordellii* at the time of our previous publication [45]. SCFA, short-chain fatty acids; H_2_S, hydrogen sulfide; BA, bile acids

We previously isolated 14 distinct bacterial taxa from *T.* *trichiura*-colonized Orang Asli (OA1 − 14) and assigned genus-species designation based on whole genome sequencing (Fig. 1) [45]. OA isolates represented six Clostridiales families: Erysipelotrichaceae, Coprobacillaceae, Clostridiaceae, Peptostreptococcaceae, Oscillospiraceae, and Lachnospiraceae. One of the Lachnospiraceae members, OA3, was a new species in the *Ruminococcus* genus based on sequence identity and was designated *Ruminococcus pangsunibacterium*. OA12 was identified as *Enterococcus* *hirae*. Although not a member of the Clostridiales order, the resistance of *Enterococci* to chemical treatment likely explains how this bacterium was retained after fractionation [46, 47]. Similar to the other OA isolates, OA12 was enriched in Malaysian microbiomes [45] and was therefore included in subsequent experiments. OA isolates were analyzed for in vitro enzymatic and metabolomic activity and then screened for their capacity to promote Treg differentiation and induce transcriptional responses in vivo. Finally, we performed correlation analyses to look for relationships between all measured variables to identify bacterial properties related to Treg differentiation or *Trichuris* hatching.

### Enzymatic profile of the OA isolates

**Fig. 2:**
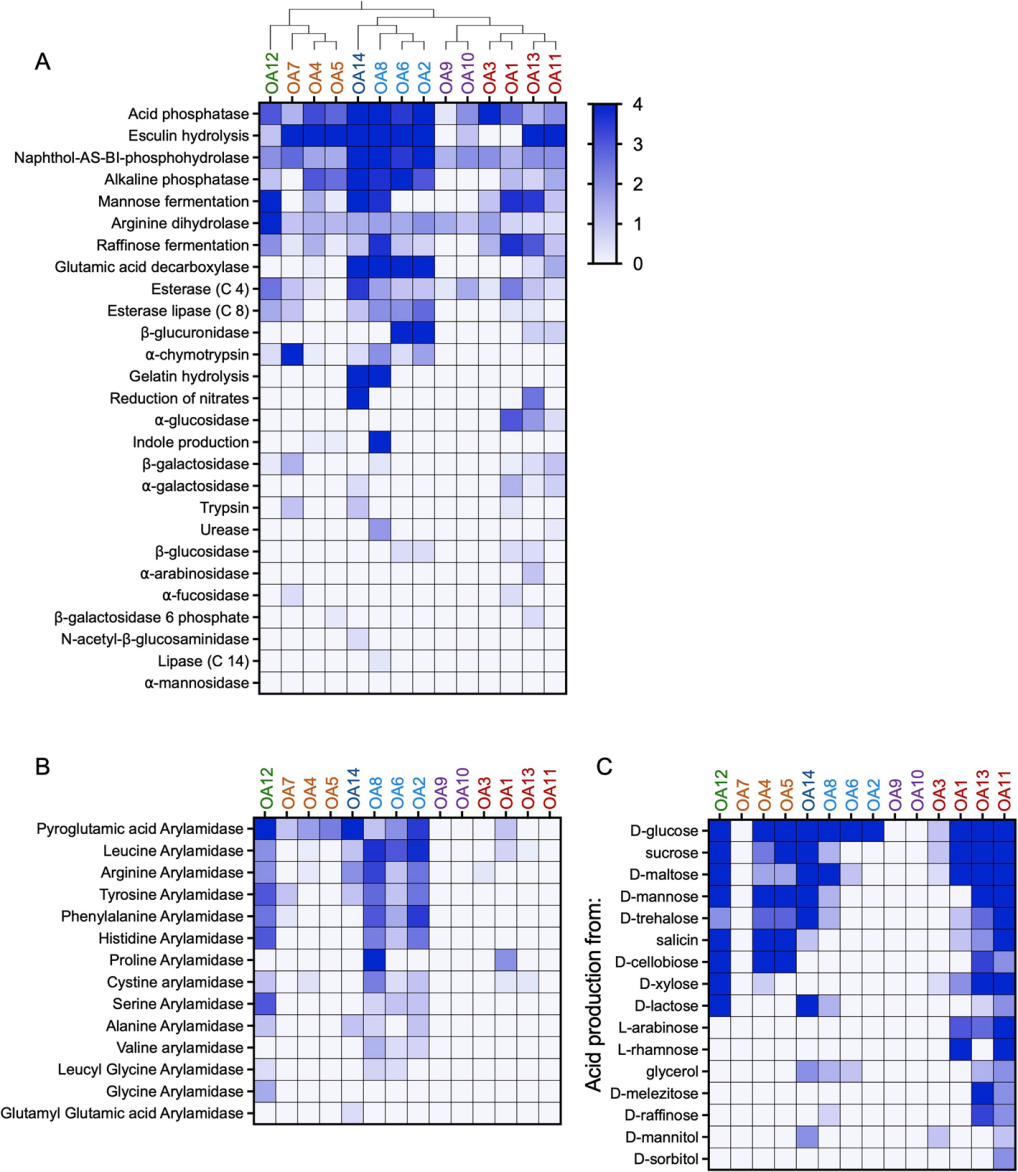
Enzymatic profile of the OA isolates. Enzyme activity (**A**), arylamidase activity (**B**), and acid production from various carbohydrates (**C**) by the OA isolates were measured using API colorimetric kits for microbial identification. Each reaction was run in at least three independent trials for each isolate, and the intensity of the reaction was assessed visually on a scale of 0 (no reaction) to 4 (strong or complete reaction). The phylogenetic tree at the top of A depicts the relationships between OA isolates

OA isolates were functionally profiled for their ability to perform 57 enzymatic reactions that represent common bacterial activities including carbohydrate metabolism. Taxonomically related isolates were more likely to share activities (Fig. 2a–c). For instance, Peptostreptococcaceae isolates (OA2, 6, and 8) and *Clostridium* *perfringens*-OA15 displayed high levels of acid phosphatase, esculin hydrolysis, naphthol-AS-BI-phosphohydrolase, and glutamic acid decarboxylase activities (Fig. 2a) and the ability to metabolize D-glucose (Fig. 2c). The Peptostreptococcaceae isolates, in addition to *E. hirae*-OA12, also had the highest arylamidase activities, both in terms of the degree of activity and the different amino acid substrates (Fig. 2b). As expected, the two *Clostridium* *innocuum* isolates OA4 and OA5 displayed similar profiles for the majority of the 57 parameters, although there were a few enzymatic reactions in which they diverged. For instance, *C. innocuum*-OA4 but not *C. innocuum*-OA5 showed mannose and raffinose fermentation activities. *E. hirae*-OA12 and *Enterocloster* species OA11 and OA13 displayed the broadest capacities to metabolize carbohydrates (Fig. 2c).

The enzymatic profiles of the Peptostreptococcaceae isolates *Romboutsia* *hominis*-OA2 and -OA6, and *Paraclostridium* *sordellii*-OA8 were furthered measured in stool from mice monocolonized with these isolates. Although many enzymes could not be measured due to high background in germ-free (GF) stool, those that could be detected reflected a similar profile as observed in vitro (Figure S1). For example, both α-chymotrypsin and β-glucuronidase activities were low in stool from GF mice and high in stool from conventional-specific pathogen-free (SPF) mice. Although stool from monocolonized mice did not produce a positive signal for α-chymotrypsin and were indistinguishable from GF-negative controls, β-glucuronidase activity revealed positive signals for *R. hominis*-OA2 and OA6 and a negative signal for *P. sordellii*-OA8, matching the pattern of enzymatic activity observed in vitro (Fig. 2a). Overall, these data indicate that OA isolates possess a broad range of metabolic activities, consistent with their taxonomic diversity.

#### OA isolates produce short-chain fatty acids and hydrogen sulfide

**Fig. 3:**
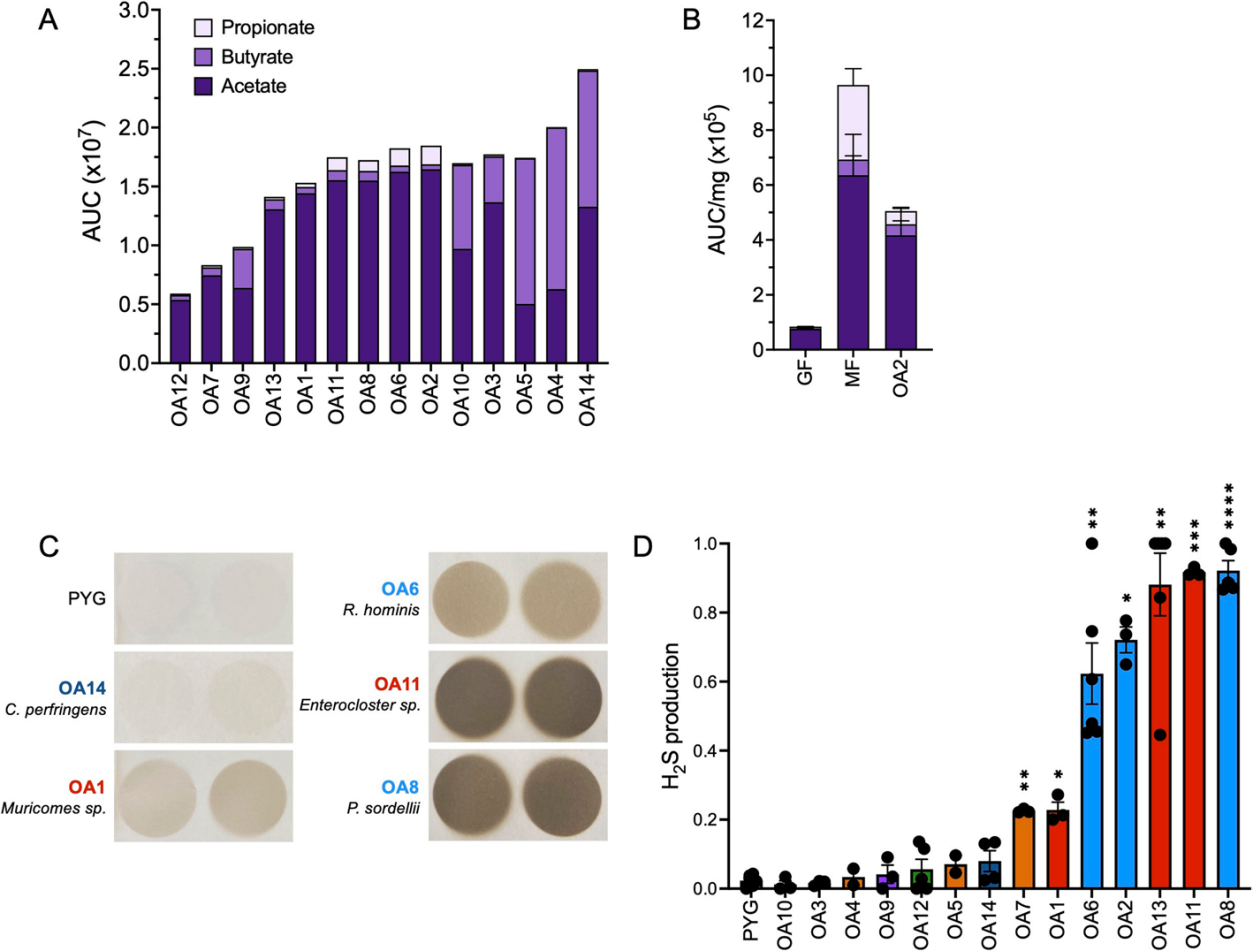
OA isolates produce short-chain fatty acids and hydrogen sulfide. **A** Short-chain fatty acid (SCFA) production in bacterial culture supernatants measured by mass spectrometry. AUC, area under curve. **B** SCFA concentration per milligram of cecal contents from germ-free (GF), minimal flora (MF), or *R. hominis*-OA2-monocolonized (OA2) mice, measured by mass spectrometry. AUC, area under curve. **C** Representative images of lead acetate paper used to detect hydrogen sulfide (H_2_S) production by the OA isolates in vitro. Darker color indicates more H_2_S produced by bacterial cultures. **D** Quantification of H_2_S production by each OA isolate, normalized to the amount of H_2_S production by media alone (“PYG”) within each experiment. Kruskal–Wallis with Dunn’s multiple comparisons test was used to compare between each group and PYG. *p < 0.05, **p < 0.01, ***p < 0.001, ****p < 0.0001

Given the link between helminth-associated microbiota and anti-inflammatory responses, we measured the production of short-chain fatty acids (SCFAs) and hydrogen sulfide (H_2_S) by the OA isolates. Bacteria ferment soluble fibers to produce short-chain fatty acids (SCFAs) which include acetate, propionate, and butyrate [48]. SCFAs have been shown to regulate a variety of immune cell types [9]. For example, they can induce Treg expansion in the gut to regulate intestinal inflammation [49–51]. Although levels varied, we detected acetate in the culture supernatant for all 14 OA isolates. *C.* *innocuum*-OA4 and -OA5 and *C. perfringens*-OA14 produced butyrate at levels similar to or greater than acetate. Propionate was not detected or negligible except for *R.* *hominis*-OA2 and -OA6, *P. sordellii*-OA8, and *Enterocloster *sp. OA11 (Fig. 3a). We validated this finding by testing cecal samples from mice monocolonized with *R. hominis*-OA2 and included GF mice as a negative control and mice colonized with a previously described synthetic minimal flora (MF) consisting of 15 bacteria [52, 53] as a positive control. As expected, we detected minimal levels of SCFAs in GF mice compared with MF mice in which all three were detected. Mice monocolonized with OA2 displayed the proportion of the three SCFAs predicted by the in vitro analysis (Fig. 3b).

Some bacteria can produce hydrogen sulfide (H_2_S), which suppresses inflammation in multiple disease models and is associated with Treg differentiation [54–59]. We found that Peptostreptococcaceae isolates OA2, 6, and 8, as well as *Enterocloster* isolates OA11 and OA13, were potent producers of H_2_S, suggesting another possible mechanism by which these bacteria could have immunomodulatory interactions with the host (Fig. 3c–d).

### Several OA isolates induce regulatory T cells in the gut

Peripherally induced Tregs (iTregs) in the gut that develop in response to microbial antigens and metabolites are distinguished by expression of the transcription factor *forkhead* *box P3* (*Foxp3*) and *RAR‐related orphan receptor γt* (*Rorγt*). These Foxp3^+^ Rorγt^+^ Tregs contribute to immune tolerance of the microbiota and suppress colitis [60–64]. iTregs can be further distinguished from thymus-derived or natural Tregs (nTregs) reactive to self-antigens by the absence of the transcription factor Helios (Ikzf2) [65]. GF mice possess low numbers of iTregs, and thus, the induction of iTregs in GF mice in response to colonization by bacteria serves as a sensitive assay to measure immunomodulatory potential of commensal species [61, 66–68].

**Fig. 4:**
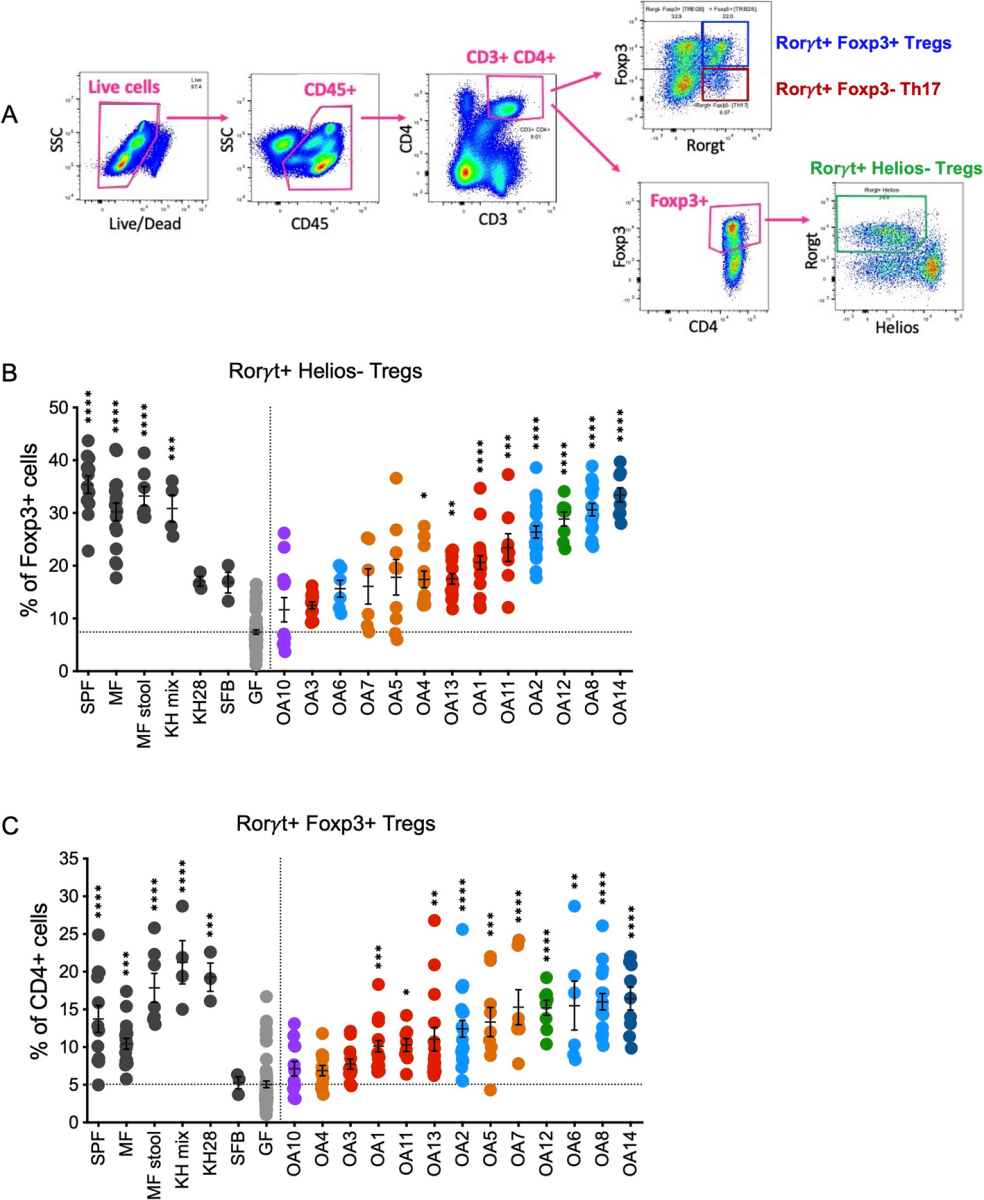
OA isolates induce iTregs in the colon. **A** Representative flow cytometry gating strategy for Rorγt^+^ Helios^−^ iTregs out of Foxp3^+^ cells, Foxp3^+^ Rorγt^+^ iTregs out of CD4^+^ cells, and Rorγt^+^ Foxp3^−^ Th17 cells out of CD4^+^ cells from colon lamina propria cells. **B** Frequencies of Rorγt^+^ Helios^−^ cells within Foxp3^+^ Tregs. **C** Frequencies of Rorγt^+^ Foxp3^+^ Tregs out of CD4^+^ T cells. SPF, specific pathogen-free; MF, minimal flora mice; MF stool, GF mice gavaged with stool from minimal flora mice; KH mix, mice gavaged with consortium of 17 Clostridia strains from Atarashi et al. (2013) [5]; KH 28, *Clostridium* *aldenense* from the KH mix; SFB, segmented filamentous bacteria; GF, germ-free. Each dot corresponds to one mouse. Each group was tested in at least two independent experiments consisting of 2–5 mice. Kruskal–Wallis with Dunn’s multiple comparisons test was used to compare between each group and GF. *p < 0.05, **p < 0.01, ***p < 0.001, ****p < 0.0001

We analyzed iTregs in the colonic lamina propria of GF mice by flow cytometry 3 to 4 weeks postoral inoculation with individual OA isolates (Fig. 4a), except *Lawsonibacter* sp. OA9 which failed to colonize GF mice in the absence of other bacteria. In addition to SPF mice and untreated GF mice that served as benchmarks, we included several controls to aid in interpretation of the quantitative data. As a positive control, we colonized mice with a mixture of 17 Clostridia isolates (KH mix) previously shown to induce Tregs in GF mice [5]. As negative controls, we used mice monocolonized with segmented filamentous bacteria (SFB), a bacterium that induces Th17 cells (Rorγt^+^ Foxp3^−^ CD4^+^ T cells) without inducing Tregs [69], and mice monocolonized with one of the KH mix strains, *Clostridium* *aldenense* (KH28), which is insufficient to induce Tregs to levels achieved with the entire KH mix consortium [5]. We also quantified Tregs in MF mice described above (mice raised in a gnotobiotic isolator containing MF) and GF mice gavaged with stool from MF mice (MF stool). Stable colonization was confirmed for all conditions based on detection of bacteria in stool (Figure S2a).

As expected, GF mice had lower frequencies of Rorγt^+^ Helios^−^ Tregs out of all Foxp3^+^cells and lower Foxp3^+^ Rorγt^+^ Tregs out of CD4^+^cells compared to SPF, MF, MF stool, and KH mix groups (Fig. 4b–c). We observed a spectrum of Treg induction by the OA isolates. The proportion of Foxp3^+^ Rorγt^+^ Tregs and Foxp3^+^ Rorγt^+^ Helios^−^ Tregs were restored to similar levels as SPF or MF mice in mice monocolonized with several of the OA isolates including *P. sordellii*-OA8, *E. hirae*-OA12, and *C.* *perfringens*-OA14. In contrast, several OA isolates such as *R.* *pangsunibacterium*-OA3 and *Lawsonibacter* *asaccharolyticus*-OA10 were similar to SFB and failed to induce significant increases in these Treg populations. Mice monocolonized with KH28 increased the frequency of Foxp3^+^ Rorγt^+^ Tregs out of CD4^+^cells but not Rorγt^+^ Helios^−^ Tregs out of Foxp3^+^cells (Fig. 4b–c). In addition to changes in the proportion of these populations, we quantified the absolute number of Tregs per colon. This analysis largely confirmed the above findings by showing that OA isolates that increased the relative frequency of Foxp3^+^ Rorγt^+^ Tregs, and Foxp3^+^ Rorγt^+^ Helios^−^ Tregs also increased the total number of these cells (Figure S2b–c).

### Treg induction by OA isolates correlates with Th17 but not colonization levels

**Fig. 5:**
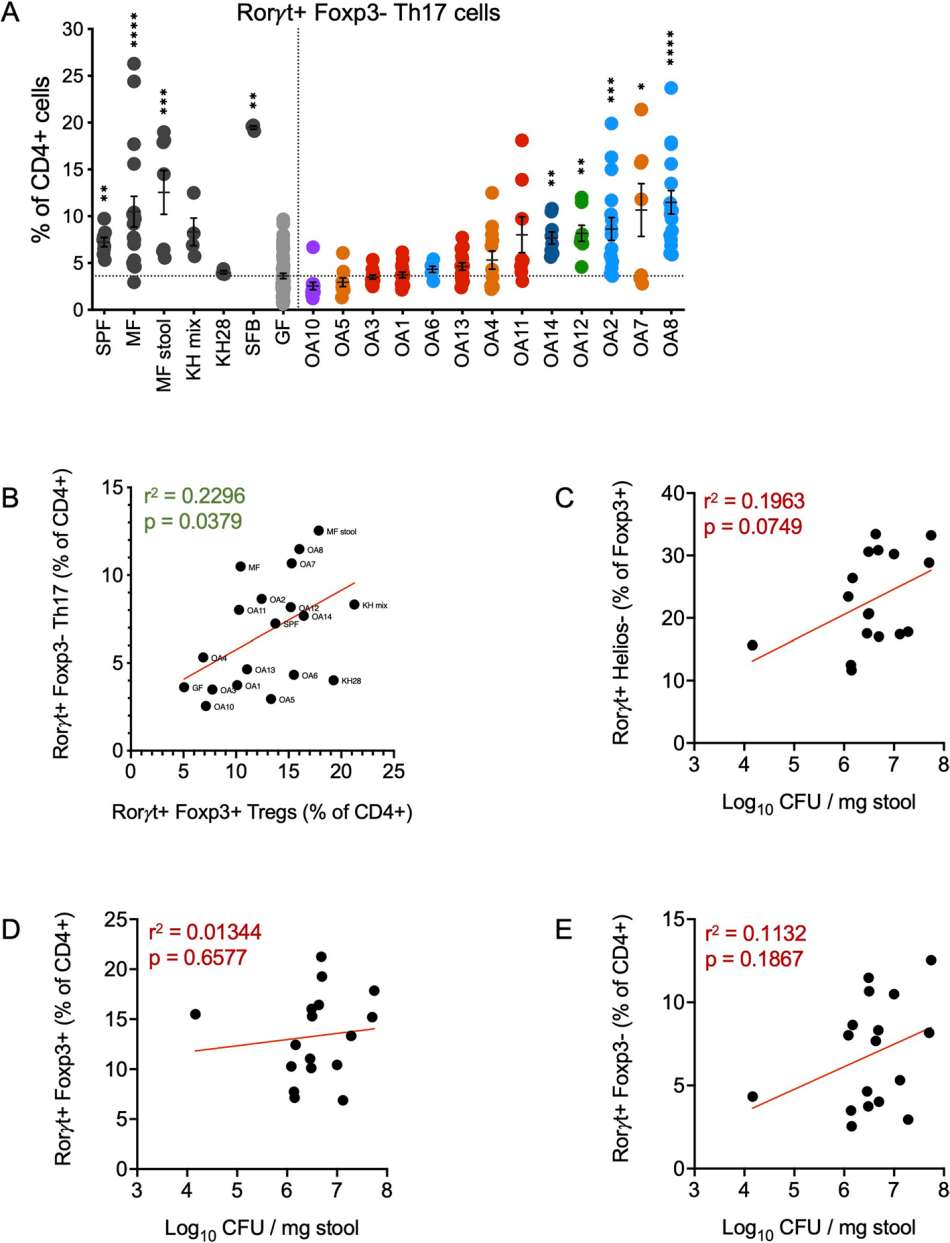
Associations between OA isolate colonization, Tregs, and Th17 cell levels. **A** Frequencies of Rorγt^+^ Foxp3^−^ Th17 cells out of CD4^+^ T cells. **B** Spearman correlation between the frequency of Foxp3^+^ Rorγt^+^ iTregs out of CD4^+^ cells and Rorγt^+^ Foxp3^−^ Th17 cells out of CD4^+^ cells induced by each bacterium or control group. **C**–**E** Spearman correlation between the bacterial burden in each mouse and the proportions of Rorγt^+^ Helios^−^ iTregs out of Foxp3^+^ cells (**C**), Foxp3^+^ Rorγt^+^ iTregs out of CD4^+^ cells (**D**), or Rorγt^+^ Foxp3^−^ Th17 cells out of CD4^+^ cells (**E**). In A, SPF, specific pathogen-free; MF, minimal flora mice; MF stool, GF mice gavaged with stool from minimal flora mice; KH mix, mice gavaged with consortium of 17 Clostridia strains from Atarashi et al. (2013) [5]; SFB, segmented filamentous bacteria; GF, germ-free. Each dot corresponds to one mouse. Each group was tested in at least two independent experiments consisting of 2–5 mice. Kruskal–Wallis with Dunn’s multiple comparisons test was used to compare between each group and GF. *p < 0.05, **p < 0.01, ***p < 0.001, ****p < 0.0001. In B–E, *r* ^2^ and *p*-values are depicted on each plot. Each dot corresponds to the average of at least two independent repeats with 2–5 mice within each experiment, and the red line depicts the linear regression slope

Given their role in mucosal immunity [70, 71], we examined whether OA isolates affect the proportion of Th17 cells (Rorγt^+^ Foxp3^−^ CD4 + T cells) (Fig. 4a) in the gut. Similar to Tregs, we found that monocolonization with OA isolates led to a range of Th17 cell levels in the colon, although none reached the extraordinarily high levels detected in mice monocolonized with SFB (Fig. 5a, S2d). Excluding SFB, we found that the proportion of Th17 cells in the colon roughly correlated with the proportion of Tregs (Fig. 5b). There were notable exceptions, such as OA6, which was a strong Treg inducer but did not increase the frequency of Th17 cells above levels seen in GF mice.

These observations raised the possibility that T-cell differentiation reflected the degree of colonization by bacteria. However, there were no significant correlations between the bacterial burden and the proportions of Tregs or Th17 cells (Fig. 5c–e), suggesting that the level of T-cell differentiation is a specific property of each bacterium. Interestingly, we found that colonization by some OA isolates increased total bile acids (BAs) detected in the stool compared with GF mice (Figure S5). Transformation of BAs by gut bacteria is associated with differentiation of Tregs and Th17 cells [72–75]. However, we did not observe a correlation between total BAs and these T-cell populations.

### Host transcriptional response to colonization by OA isolates

*R. hominis*-OA6 is unable to induce Tregs despite its taxonomic relationship to the other two Peptostreptococcaceae isolates, the Treg-inducing *R. hominis*-OA2 and *P. sordellii*-OA8. To better characterize the host response to the three Peptostreptococcaceae isolates, we performed RNAseq on colonic tissue from mice monocolonized with these three bacteria, as well as GF mice (Figure S3). Principal coordinate analysis (PCA) showed that the four conditioned mainly separated on the PC1 axis, with *R. hominis*-OA2 the farthest from GF controls and *R. hominis*-OA6 and *P. sordellii*-OA8 in-between (Figure S3a). As expected, differential gene expression analysis showed that mono-colonization with any of these bacteria led to an enrichment of immunoglobulin (Ig) transcripts, a marker of B-cell and antibody responses to the presence of bacteria (Figure S3b–d). However, certain aspects of the B-cell response may be OA isolate-specific. *Pax5* and *Ms4a1* (CD20) are both involved in B-cell differentiation [76, 77], and their transcripts are selectively induced by *R. hominis*-OA2 and *P. sordellii*-OA8 (Figure S3f, l–o). In contrast, *R. hominis*-OA6 monocolonized mice exhibited higher expression of *Postn* and *Cyp26b1* compared to OA2 and OA8. *Postn* encodes periostin, a secreted extracellular matrix protein involved in tissue development and regeneration [78], while *Cyp26b1* encodes a cytochrome P450 protein involved in drug metabolism and lipid synthesis, which has also been implicated in the differentiation and function of CD4 + T cells, including iTregs [79].

*Foxp3* expression was consistent with the Treg induction observed in vivo by these isolates compared to GF (Figure S3g). Furthermore, *Foxp3* expression was correlated with Arhgap22 (Figure S4), one of the top genes downregulated in monocyte-derived alternatively activated macrophages (AAMs) under Treg-inducing conditions [80]. This observation suggests a potential relationship between AAMs and Treg induction during colonization by helminth-associated bacteria.

### Correlations between properties of OA isolates

**Fig. 6:**
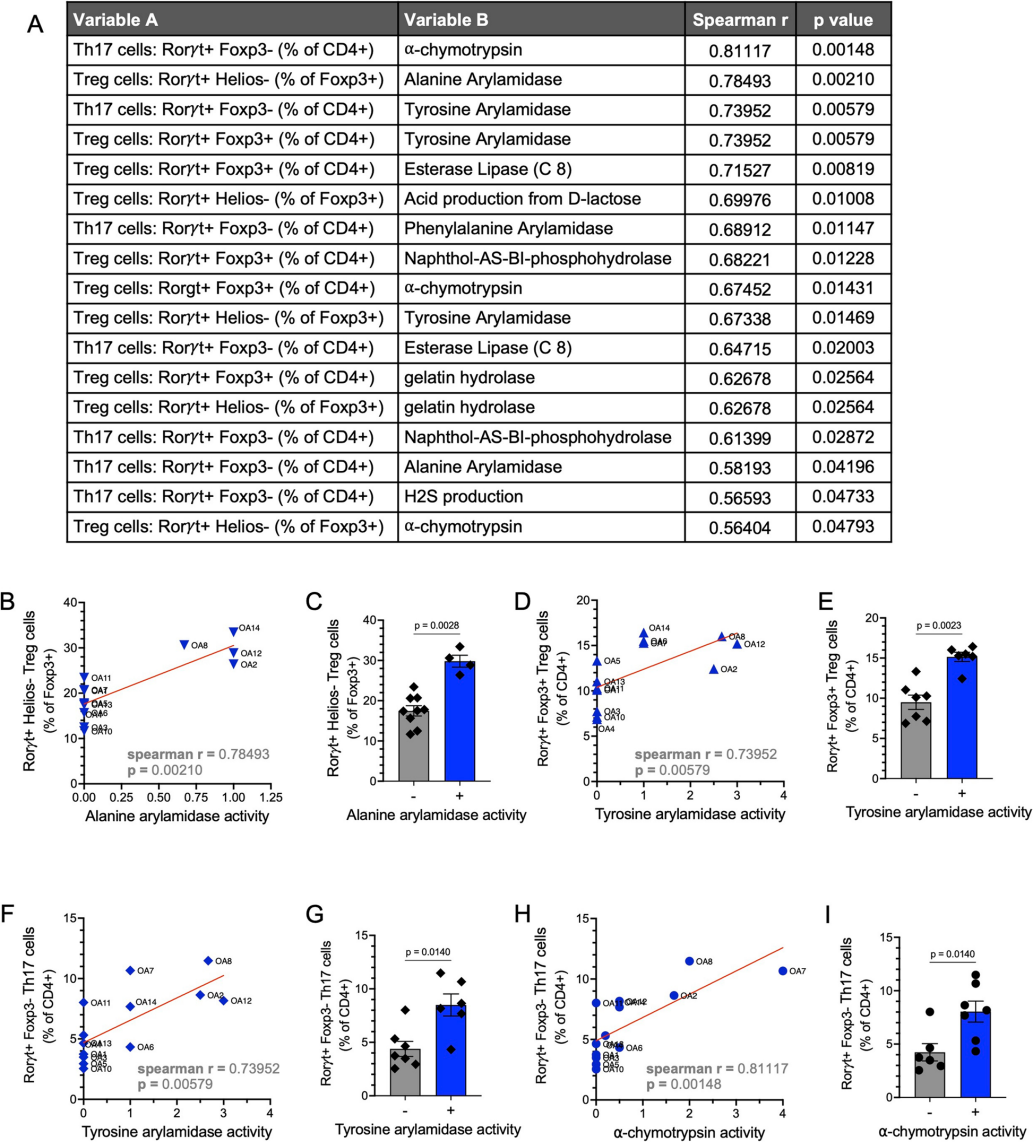
Treg and Th17 cell induction by the OA isolates is correlated with enzymatic activities by bacteria. **A** Spearman *r* and *p*-values corresponding to significant correlations between the frequency of induced Treg and Th17 cells and other measured variables of the OA isolates. **B** Graph displaying relationship between alanine arylamidase activity and induction of Rorγt^+^ Helios^−^ Foxp3^+^ iTregs for the OA isolates. **C** Quantification of Rorγt^+^ Helios^−^ Foxp3^+^ iTregs comparing OA isolates with or without alanine arylamidase activity. **D** Graph displaying relationship between tyrosine arylamidase activity and induction of Rorγt^+^ Foxp3^+^ Tregs for the OA isolates. **E** Quantification of Rorγt^+^ Foxp3^+^ Tregs comparing OA isolates with or without tyrosine arylamidase activity. **F** Graph displaying relationship between tyrosine arylamidase activity and induction of Rorγt^+^ Foxp3^−^ Th17 cells for the OA isolates. **G** Quantification of Rorγt^+^ Foxp3^−^ Th17 cells comparing OA isolates with or without tyrosine arylamidase activity. **H** Graph displaying relationship between ⍺-chymotrypsin activity and induction of Rorγt^+^ Foxp3^−^ Th17 cells by the OA isolates. **I** Quantification of Rorγt^+^ Foxp3^−^ Th17 cells comparing OA isolates with or without ⍺-chymotrypsin activity. In **B**, **D**, **F**, **H**, Spearman *r* and *p*-values are depicted on each plot in. Each dot corresponds to the average value per OA isolate, and the red line depicts the linear regression slope. In **C**, **E**, **G**, **I**, Each dot corresponds to the average value per OA isolate. Bar graphs show mean + / − SEM. Mann–Whitney test with *p*-values depicted on the bar graphs

To identify relationships between the properties measured for each OA isolate, we performed a Spearman correlation analysis between all measured variables (Figure S6). This analysis identified several enzymatic activities that were correlated with T-cell differentiation (Fig. 6a). Induction of Tregs was positively correlated with alanine arylamidase and tyrosine arylamidase activity by bacteria (Fig. 6a–e). Th17 cell induction was also positively correlated with tyrosine arylamidase activity and additionally associated with ⍺-chymotrypsin activity (Fig. 6a, f–i). Alanine arylamidase and tyrosine arylamidase have not previously been linked to Treg or Th17 cell biology. ⍺-Chymotrypsin is a member of the serine protease family and has been shown to have antibacterial properties [81] and is also made by pathogenic bacteria in the *Vibrio* genus [82]. Whether this correlation reflects a role of ⍺-chymotrypsin in Th17 cell induction or merely reflects the pro-inflammatory capacity of bacteria that produce this protease and induce Th17 cells through other mechanisms remains to be determined.

To quantify how bacterial properties explain the variance in lymphocyte differentiation, we performed a distance-based redundancy analysis (dbRDA) using the characteristics found to be most significantly related with Treg or Th17 induction according to the Spearman correlation analysis and LASSO regression (Figure S7a–b, Table S2). The analysis revealed that these variables together contribute to the variance of responses in Treg and Th17 induction, with the variation in alanine arylamidase production accounting for 41.95% of the variance, and all other variables accounting for less than 20% of the variance each (Figure S7c).

**Fig. 7:**
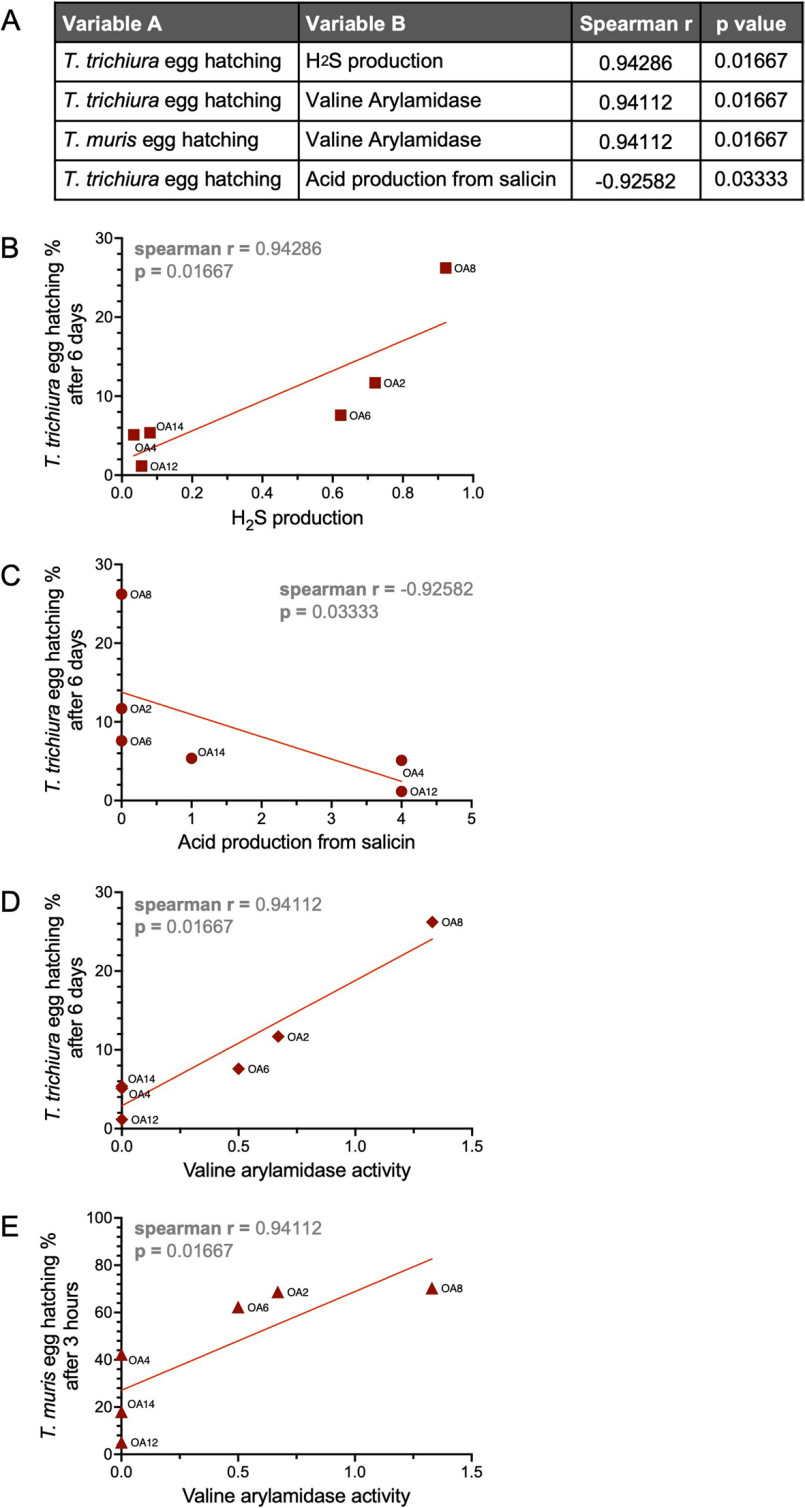
H_2_S, salicin acidification, and valine arylamidase are associated with *Trichuris* *egg* hatching by bacteria. **A** Spearman *r* and *p*-values corresponding to significant correlations between *Trichuris* egg hatching and other measured variables of the OA isolates. **B** Relationship between H_2_S production by the OA isolates and *T.* *trichiura* egg hatching rate. **C** Relationship between acid production from salicin by the OA isolates and *T.* *trichiura* egg hatching rate. **D** Relationship between valine arylamidase activity by the OA isolates and *T.* *trichiura* egg hatching rate. **E** Relationship between valine arylamidase activity by the OA isolates and *T.* *muris* egg hatching rate. Spearman *r* and *p*-values are depicted on each plot. Each dot corresponds to the average value per OA isolate, and the red line depicts the linear regression slope

We also incorporated data from our previous study in which we quantified the ability of the OA isolates to mediate hatching of eggs from *T.* *trichiura* and the mouse parasite *Trichuris muris* [45]. Hatching of *T.* *trichiura* but not *T.* *muris* was positively correlated with H_2_S production and negatively correlated with salicin acidification by the OA isolates (Fig. 7a–c). H_2_S, in addition to being involved in a wide variety of physiological processes, has been shown to promote hatching of eggs from *Ascaris suum*, a helminth that infects pigs [83]. Salicin utilization by bacteria was previously shown to be toxic to nematodes in soil [84], although these experiments were not done with *Trichuris* species. Our finding that these bacterial properties are significantly related to egg hatching of only the human whipworm could provide insight into mechanisms that may differentiate dependencies of *T.* *trichiura* and *T.* *muris* on bacterial factors for hatching. Valine arylamidase activity was significantly correlated with hatching of both *T.* *muris* and *T.* *trichiura *eggs (Fig. 7a, d–e). Valine arylamidases were among the proteases found to be part of the excretory/secretory products of *Contracaecum rudolphii*, a nematode that infects mammals and birds [85]. This observation suggests a role for this protease in *Trichuris* egg hatching. Finally, there was no correlation between the *Trichuris* egg hatching rate and iTreg and Th17 cell-inducing ability of the OA isolates (Figure S8), indicating that bacterial taxa present in the helminth-colonized microbiome are either potent inducers of egg hatching or T-cell differentiation, but not necessarily both.

## Discussion

Our comprehensive enzymatic profiling of OA isolates coupled with analysis of SCFAs and H_2_S production revealed a broad range of metabolic activities that are associated with host physiology, including immunity. Consistent with this observation, several OA isolates increased the number and proportion of colonic iTregs when introduced into GF mice, comparable to levels achieved upon colonization with the mixture of 17 Clostridia strains used as our benchmark. SCFAs were previously associated with the ability of the Clostridia mixture to induce Tregs [5, 6]. We did not observe a correlation between SCFA production and iTreg induction by the OA isolates, indicating that SCFAs are not sufficient to distinguish Treg-inducing OA isolates. Subsequent to the initial characterization of the Clostridia mixture, other metabolites such as bile acids have been shown to mediate Treg differentiation in the presence of intestinal bacteria [86], which may contribute to Treg differentiation induced by OA isolates. Although there was not a significant relationship with iTreg or Th17 induction when considering all the OA isolates, it is possible that SCFAs and bile acids distinguish Treg inducers within specific taxonomic groups. Isolation of additional strains belonging to groups of interest such as Peptostreptococcaceae would help address this important question.

Alternatively, it is possible that previously unknown factors are involved. For example, we identified strong positive correlations between Treg induction and alanine arylamidase and tyrosine arylamidase activities that warrant further study. Our observation that three different species, *R. hominis*-OA2, *P. sordellii*-OA8, and *E. hirae*-OA12, consistently exhibited the highest Treg and Th17 cell induction, as well as arylamidase activity, suggests a predictive potential for these enzymes in identifying bacteria with immunomodulatory capacity.

Although *R. hominis*-OA2, *R. hominis*-OA6, and *P. sordellii*-OA8 are all members of the Peptostreptococcaceae family, *R. hominis*-OA6 fails to induce Th17 cells and Rorγt^+^ Helios^−^ Tregs. We performed RNAseq to determine if the host response to these closely related isolates was different. Indeed, the T-cell inducers *R. hominis*-OA2 and *P. sordellii*-OA8 increased expression of the B-cell differentiation and activation factors *Pax5* and *Ms4a1* while *R. hominis*-OA6 did not. Overall, this is one of the few studies that have functionally profiled individual bacterial isolates from understudied populations beyond inferring properties from genomic data.

The ability of some OA isolates to induce both Tregs and Th17 cells is consistent with the co-regulation of these cell types. Thus, a model in which helminth-associated bacteria inhibit inflammatory diseases by shifting the balance towards immune tolerance may be too simplistic. Although our approach allowed us to examine properties of individual bacteria in isolation and reduce variables, it would be interesting to determine whether the presence of *Trichuris* alters the properties of OA isolates or vice versa. Along these lines, we noted correlations between *Trichuris* egg hatching as determined in our prior study and H_2_S production and valine arylamidase activity. Arylamidases catalyze the release of N-terminal amino acids from peptides. Recently, we discovered that protease inhibitors block bacteria-mediated egg hatching, most likely by interfering with surface protein structures necessary for contact between bacteria and egg [87]. Therefore, we speculate that valine arylamidase production is necessary for structural properties of the bacteria that mediate hatching.

In addition, the helminth-associated Peptostreptococcaceae isolates *R. hominis*-OA2, *R. hominis*-OA6, and *P. sordellii*-OA8 had high glutamic acid decarboxylase (GAD) activity which is lacking in *E. hirae*-OA12, an isolate that fails to induce *T.* *muris* and *T.* *trichiura* egg hatching (Fig. 2A) [45]. GAD catalyzes the formation of the neurotransmitter gamma-aminobutyric acid (GABA), and bacterially produced GAD and GABA have been shown to act on the free-living nematode *Caenorhabditis* *elegans* [88]. Such mechanisms involved in *Trichuris* egg hatching may be unrelated to host responses because we did not find a significant correlation between the ability of specific OA isolates to induce *Trichuris* egg hatching and T-cell differentiation (Figure S8). It is possible that *Trichuris* worms may benefit from lower burdens within the mammalian gut to prevent induction of a strong type 2 response that would lead to its expulsion. Inoculation of mice with low numbers of *T. muris* eggs leads to chronic infection, while a high inoculum induces a stronger Th2 response leading to expulsion [35, 36, 89]. Hence, it may be of greater benefit in some situations to have bacteria inducing a more anti-inflammatory response with more iTregs while being poor inducers of egg hatching.

An important limitation of this study is the difficulty in linking the properties of the different Clostridiales isolates to Treg induction and helminth colonization. Genetic manipulation for Clostridiales species is exceedingly challenging and has not been attempted for many of the species represented in our study. In addition, *Trichuris* species are not genetically amenable. However, genetic manipulation of *C. perfringens* and *P.* *sordellii* has been successful by others [90], and future work using these systems could yield important insights on bacterial properties important for Treg induction and helminth colonization by these species.

In conclusion, our findings describe the functional properties of bacteria isolated from helminth-colonized individuals in Malaysia and identify correlates of Treg induction and *Trichuris* egg hatching. Although our analyses focused on a finite set of parameters and bacterial taxa, they unveil key features of these specific helminth-associated bacteria that can be targeted for deeper analysis in future studies. As more bacteria from individuals in understudied populations become available for study along with their full genomes, it will be important to determine the extent to which these functional properties are represented in related taxa, which will enable us to infer how the selective presence of these bacteria in certain populations can impact their health status.

## Materials and methods

### Gnotobiotic mice

Germ-free (GF) C57BL/6 J were bred and maintained in flexible-film isolators at the New York University Grossman School of Medicine Gnotobiotics Animal Facility. The absence of fecal bacteria was confirmed monthly by evaluating the presence of 16S DNA in stool samples by qPCR as previously described [91]. Minimal flora (MF) mice harboring the consortium of 15 bacteria described in [52] were kept in a separate isolator. For inoculation with bacteria, GF mice were housed in Bioexclusion cages (Tecniplast) with access to sterile food and water. An equal amount of male and female mice 6–8 weeks of age was used for all experiments. All animal studies were performed according to protocols approved by the NYU Grossman School of Medicine Institutional Animal Care and Use Committee. The RNAseq experiment was conducted using C57Bl/6NTac mice bred and housed at NIAID’s gnotobiotic animal facility with access to standard sterile chow and water ad libitum.

### Bacterial strains

OA isolates were previously described [45]. The consortium of 17 Clostridia isolates (KH mix) including *Clostridium* *aldenense* (KH28) was kindly provided by K. Honda (RIKEN Center for Integrative Medical Sciences, Japan) [5]. All bacteria were cultured under anaerobic conditions in an anaerobic chamber (Coy Labs). Frozen glycerol stocks (30% glycerol) of all bacteria were prepared. Glycerol stocks of the OA isolates were streaked onto BRU agar plates (Anaerobe Systems) and incubated anaerobically for 48 h at 37 °C. PYG broth (Anaerobe Systems) inoculated with single colonies was grown at 37 °C. OA1, 2, 4, 5, 6, 8, 11, 12, 13, 14, the KH mix, and KH28 were grown for 24 h. OA3, 7, 9, and 10 required 3 days to reach similar turbidity. To quantify colony-forming units, we performed serial dilutions of liquid culture in sterile PBS and plated on BRU agar plates*.* Segmented filamentous bacteria (SFB) were kindly provided by D. Littmann (NYU Grossman School of Medicine) [69] in the form of stool from GF mice monocolonized with SFB. SFB burden was confirmed in stool by qPCR as described in [69].

### Enzyme activity assays

OA isolates were grown anaerobically for 24 h or 3 days, prepared as per manufacturer’s instructions, and inoculated onto test strips from API® ZYM, API® Rapid ID 32A, and API® 20A Microbial Identification Kits (bioMerieux). Test strips were incubated at 37 °C for either 4 h or 24 h and at either aerobic or anaerobic conditions depending on the manufacturer’s instructions, after which enzyme reactions were assessed visually based on colorimetric changes.

To test the enzymatic activity of bacteria in fresh stool from monocolonized mice, we modified the methods reported in *Rada *et al*. *[92]. Approximately, 50 mg of fresh feces was collected from each mouse in the groups colonized with OA strains, and the GF control group, as well as from two adult C57Bl/6 mice with a standard microbiota. Feces from GF mice were used as negative controls, and feces from the adult C57Bl/6 mice were used as positive controls. The feces from each mouse were resuspended in 10 mL of sterile saline and centrifuged for 10 min at 4000 rcf. The supernatant was discarded, and the pellet was resuspended in 1 mL of saline. For the API ZYM kit, 90 µL of the resuspended pellets was dispensed into wells 3, 4, 6, 10, 11, 12, and 15. For the Rapid ID 32A kit, 55 µL of the final fecal suspension was dispensed into the wells for the TyrA and AlaA tests. The enzymatic reactions were carried out according to the instructions for each kit in incubators at 37 °C. For the Rapid ID 32A kit, the incubator was inside an anaerobic chamber (Vinyl Anaerobic Chamber, Coy Laboratory Products, MI, USA), and the kits were always handled inside the chamber. The atmosphere of the anaerobic chamber was 5% H2, 5% CO2, and 90% N2. After 3.5–4 h, the test strips were taken out of the incubators and brought to the bench in aerobic conditions. Photos were immediately taken of the assays, and ~ 30 µl of the reaction volume was pipetted onto parafilm to facilitate visual side-by-side comparisons of the reactions’ outcome between the different samples.

### Quantification of SCFAs using LC–MS

Bacterial cultures of OA isolates were spun down at 14,000 g for 5 min at 4 °C, and supernatant was collected and stored at − 80 °C. Cecal contents were harvested from GF mice, MF mice, or mice monocolonized with OA2 and stored at − 80 °C. For the measurement of SCFAs in bacterial liquid culture, a 10-µL aliquot of the culture was mixed with 190 µL of short-chain fatty acids (SCFAs) derivatization solution (1-mM 2,2′-dipyridyl disulfide, 1-mM triphenylphosphine, and 1-mM 2-hydrazinoquinoline dissolved in acetonitrile). For the measurement of SCFAs in cecal contents, ~ 10 mg of cecal sample was resuspended in 50 µL of 50% MeOH (in H2O) and vortexed for 10 min (some beads were added to disperse the cecal material). Then the mixture was spun down, and 10 µL of supernatant was mixed with 190-µL SCFAs derivatization solution.

For both bacterial liquid culture and cecal content samples, the resulting mixtures were vortexed and incubated at 60 °C for 1 h. The mixture was centrifuged at 21,000 × g for 20 min, and the supernatant was analyzed using an Agilent 1290 LC system coupled to an Agilent 6530 quadrupole time-of-flight (QTOF) mass spectrometer with a 130 Å, 1.7 μm, and 2.1 mm × 100 mm ACQUITY UPLC BEH C18 column (Waters). We used the following solvent system: (A) H2O with 0.1% formic acid and (B) methanol with 0.1% formic acid. A total of 1 µL of each sample was injected, and the flow rate was 0.35 mL/min with a column temperature of 40 °C. The gradient for HPLC–MS analysis was as follows: 0–6.0 min, 99.5–70.0% A; 6.0–9.0 min, 70.0–2.0% A; 9.0–9.4 min, 2.0% A; 9.4–9.6 min, 2.0–99.5% A. Peaks were assigned by comparison with authentic standards.

### Hydrogen sulfide production assay

In a 24-well plate, 10 µl of 24-h or 3-day bacterial culture was added to 1 mL of sterile PYG media. Each plate was covered with a piece of lead acetate paper cut to fit the 24-well plate, followed by the plate lid over the lead acetate paper. After 24 h, photos were taken to quantify darkening of the lead acetate paper using ImageJ.

### Quantification of Treg and Th17 cell induction

Male and female germ-free C57BL/6 J mice were monocolonized at 6–8 weeks of age by oral gavage with ~ 1 × 10^7^ colony-forming units (CFU) of indicated bacteria. A total of 21–28 days later, mice were euthanized, and the colon and cecum were harvested. For single cell suspension, colonic and cecal tissues were flushed with HBSS (Gibco), fat and Peyer’s patches were removed, and the tissue was cut into 5–6 pieces. Tissue bits were incubated first with 20 mL of HBSS with 2% HEPES (Corning), 1% sodium pyruvate (Corning), 5-mM EDTA, and 1-mM dithiothreitol (Sigma-Aldrich) for 15 min at 37 °C with shaking, and then with new 20 mL of HBSS with 2% HEPES, 1% sodium pyruvate, 5-mM EDTA for 10 min at 37 °C with shaking. Tissue bits were washed in HBSS + 5% FCS, minced, and then enzymatically digested with collagenase D (0.5 mg/mL, Roche) and DNase I (0.01 mg/mL, Sigma-Aldrich) for 30–45 min at 37 °C with shaking. Digested solutions were passed through a 70-mm cell strainer (BD), and cells were subjected to gradient centrifugation using 40% Percoll (Sigma-Aldrich).

Surface and transcription factor staining was performed per manufacturer’s instructions in PBS + 2% FBS for 20 min on ice. Zombie Aqua Fixable Viability Kit (Biolegend) was used to exclude dead cells. Surface markers were stained with anti-CD45 Pacific Blue, anti-CD3 FITC, and anti-CD4 APC-Cy7 from BioLegend. For intracellular staining of transcription factors, cells were permeabilized with the eBioscience Foxp3/Transcription Factor Staining Buffer Set (Thermo Fisher Scientific) at room temperature for 30 min, and then stained with anti-Foxp3 APC and anti-Rorγt PE from Invitrogen, and anti-Helios PE-Cy7 from BioLegend. Samples were acquired on the CytoFLEX analyzer (Beckman Coulter) and analyzed using FlowJo 10.8.1.

## RNAseq

Twelve germ-free mice (8 males, 4 females) of 21 weeks of age and a C57Bl/6NTac background were used for the monocolonization experiments with Orang Asli bacterial strains. Bacterial strains OA02, OA06, and OA08 were cultured in Columbia agar plates with 5% sheep blood. The identity of the strains was confirmed by Sanger sequencing of the 16S rRNA genes from single colonies: OA02, OA06, and OA08. Single colonies from each plate were picked and cultured in liquid media for 24 h. After which, aliquots were made for each strain in 20% glycerol in sterile containers. These aliquots were stored at − 80 °C and defrosted on the day mice were inoculated with the OA strains.

Germ-free mice were split in four groups. Each group consisted of two males and one female. Before colonizing mice with the OA strains, fresh feces from all mice were screened for bacterial contamination using a flow cytometer, which confirmed their germ-free status. One group of mice remained germ-free (two males, one female), while each of the other three groups was inoculated with one OA strain by oral gavage with 200 µL of the corresponding liquid culture. Five minutes before inoculation, mice were administered 200 µL of a sterile solution of sodium bicarbonate (Sodium Bicarbonate Injection USP, 8.4%, Hospira Inc., NDC 0409–6625-22) to reduce the acids in the stomach of mice and enhance the survival of the gavaged bacteria.

Eight weeks after mice were monocolonized with the OA strains, fecal samples were collected from all groups to measure the bacterial load and verify the germ-free status of the negative control group. The bacterial load was measured in a flow cytometer by counting fecal bacteria stained with a fluorescent DNA marker (Table S1). The total DNA stain (SYBR Green I, ThermoFisher Scientific, Waltham, MA, USA) enabled distinguishing bacteria from fecal debris. Once we verified that the negative control was germ-free, we euthanized the mice and collected cecal tissue for total RNA extraction. Using a biopsy puncher with a diameter of 8 mm, we collected circular tissue pieces from the cecum of mice and preserved them in DNA/RNA Shield (Zymo Research Corporation, Irvine, CA, USA) at − 80 °C. Total RNA was extracted using a kit (Quick-RNA MiniPrep Plus, Zymo Research Corporation). Because the tissue samples were not homogenized, tissues were treated overnight with 2X proteinase K from the kit and constant tumble mixing until tissues were dissolved. The tissue digest was taken through the protocol provided by the manufacturer, including a step for the enzymatic degradation of DNA. To validate the extraction process, the nucleic acid concentration in the eluates was measured in a spectrophotometer. Aliquots were sent to a facility (Sequencing Facility, Frederick National Laboratory for Cancer Research) for the sequencing of 150-bp long paired-end reads in a NextSeq 2000 P2 platform using Illumina® Stranded Total RNA Prep and ligation with Ribo-Zero Plus.

RNA-seq results were processed using the R package DESeq2 to obtain variance stabilized count reads, fold changes relative to specific condition, and statistical *p*-value. Analysis of the OA isolates focused on differentially expressed genes (DEGs), defined as the genes with an absolute fold change relative to specific condition > 1 and an adjusted *p*-value < 0.05. DEGs were visualized using R packages ggplot2 and ComplexHeatmap.

### Quantification of bile acids in stool samples

A total of 10–15 mg of stool was homogenized in cold 100% ethanol at a final concentration of 10 mg/mL using zirconia/silica 1.0-mm beads. Homogenates were spun down at 21,000 g for 3 min at 4 °C, and 450 µL of the supernatant was transferred to a clean 1.5-mL tube. The sample was then dried down in a SpeedVac before reconstituting in ultrapure water. Bile acid concentration in each sample was quantified using the Bile Acid Assay Kit (Sigma no. MAK309) following the manufacturer’s instructions and the SpectraMax M3 Multi-Mode Microplate Reader with SoftMax Pro 6.5.1. software.

### Distance-based redundancy analysis (dbRDA)

Enzyme production levels were normalized, and one enzyme (⍺-mannosidase) that had zero production for all isolates was removed, for further statistical analyses. Least absolute shrinkage and selection operator (LASSO) regression was used as a feature selection method of enzymatic production. Enzymes that contributed significantly to prediction of Treg and Th17 induction and enzymes that had significant Spearman correlation coefficients were chosen as explanatory variables for dbRDA. Both analyses were conducted using R version 4.2.2.

### Statistical analysis

For in vitro and in vivo experiments, the number of repeats per group is annotated in corresponding figure legends. Significance for all experiments was assessed using GraphPad Prism software. Specific tests are annotated in corresponding figure legends. *p*-values correlate with symbols: ns or no symbol = not significant, *p < 0.05, **p < 0.01, ***p < 0.001, and ****p < 0.0001.

### Supplementary Information

**Additional file 1: Figure S1.** Enzymatic profile of the OA isolates in vivo. Representative images of α-chymotrypsin and β-glucuronidase enzymatic activity measured in fresh stool from mice monocolonized with the indicated OA isolates. GF, germ-free; SPF, Specific pathogen free. **Figure S2.** Bacterial burden and absolute numbers of Treg and Th17 cells induced by the OA isolates. (A) Colony forming units (CFUs) per mg of stool from mice colonized with indicated bacteria on the day cells were collected for analysis by flow cytometry. Each dot corresponds to one mouse. (B-D) Absolute numbers of Rorγt^+^ Helios^−^ Foxp3^+^ Tregs (B), Rorγt^+^ Foxp3^+^ Tregs (C) and Rorγt^+^ Foxp3^−^ Th17 cells (D) in the colon lamina propria of mice colonized with the indicated bacteria. SPF, Specific pathogen free; MF, Minimal flora mice; MF stool, GF mice gavaged with stool from Minimal flora mice; KH mix, Mice gavaged with consortium of 17 Clostridia strains from Atarashi et al., 2013; SFB, Segmented filamentous bacteria; GF, Germ-free. Each dot corresponds to one mouse. Each group was tested in at least 2 independent experiments consisting of 2–5 mice. Kruskal–Wallis with Dunn’s multiple comparisons test was used to compare between each group and GF in B-D. *p < 0.05, **p < 0.01, ***p < 0.001, ****p < 0.0001. **Figure S3.** Host transcriptional response to colonization by OA isolates. (A) PCA plot showing the overall transcriptional response (RNAseq) of cecal cells from GF mice and mice monocolonized with OA2, OA6 and OA8. Each dot corresponds to one mouse. (B-F) Heat maps of differentially expressed genes between pairs of groups. Genes with base mean > 50 and absolute value of logFC ≥ 2 are shown. (G-O) Expression of indicated genes between GF controls and monocolonized groups. Each dot represents one mouse. Bar graphs show mean ± SEM. Kruskal–Wallis with Dunn’s multiple comparisons test was used to compare between all groups. *p < 0.05. **Figure S4.** Genes correlated with *Foxp3* expression. (A) Scatter plot of transcripts correlated with Foxp3 expression. **Figure S5.** Monocolonization with a subset of OA isolates increases total bile acids in stool. Quantification of total bile acids per g of stool from mice monocolonized with the indicated bacteria. PBS, PBS as a technical control; GF, germ-free mouse; MF, minimal flora mouse. Kruskal–Wallis with Dunn’s multiple comparisons test was used to compare between each group and GF. **p < 0.01, ****p < 0.0001. **Figure S6.** Spearman correlation matrix depicting relationships between OA isolate properties. Heatmap showing Spearman correlations between all measured variables for the OA isolates including Treg and Th17 cell induction, *Trichuris* egg hatching, and production of SCFAs, BAs, H_2_S, and enzymes. Variables with insufficient data points to complete the correlation analysis were removed from the heatmap. **Figure S7.** dbRDA analysis to determine effect size of bacterial enzyme production on lymphocyte differentiation induced by the OA isolates. (A) Enzyme production data used to perform the distance-based redundancy analysis (dbRDA) and statistical significance of the F statistic for each variable. (B) PCA biplot depicting dbRDA results for Treg and Th17 cell induction and enzyme production. (C) Proportion of variance explained by 10 significant variables on the variance of Treg and Th17 cell induction. **Figure S8.** Treg and Th17 cell induction is not correlated with Trichuris egg hatching ability by the OA isolates. (A) Relationship between *T. muris* egg hatching rate and the induction of Rorγt^+^ Foxp3^+^ Tregs for the OA isolates. (B) Relationship between *T. muris* egg hatching rate and the induction of Rorγt^+^ Helios^−^ Foxp3^+^ Tregs for the OA isolates. (C) Relationship between *T. muris* egg hatching rate and the induction of Rorγt^+^ Foxp3^−^ Th17 cells for the OA isolates. (D) Relationship between *T. trichiura* egg hatching rate and the induction of Rorγt^+^ Foxp3^+^ Tregs for the OA isolates. (E) Relationship between *T. trichiura* egg hatching rate and the induction of Rorγt^+^ Helios^−^ Foxp3^+^ Tregs for the OA isolates. (F) Relationship between *T. trichiura* egg hatching rate and the induction of Rorγt^+^ Foxp3^−^ Th17 cells for the OA isolates. Spearman r and p values are depicted on each plot. Each dot corresponds to the average value per OA isolate and the red line depicts the linear regression slope.

**Additional file 2: Table S1.** Fecal bacterial loads of mice in the study after ~ 8 weeks of inoculation. Germ-free mice (GF), mice colonized with OA02, OA06, and OA08 strains, and standard laboratory mice with a full microbiota (C57BL/6). Bacterial load was measured in a flow cytometer by counting fecal bacteria stained with a fluorescent DNA marker. Bacterial load is almost two orders of magnitude larger in monocolonized mice than in GF mice, and three orders of magnitude larger in standard mice than in germ-free mice.

**Additional file 3: Table S2.** Least absolute shrinkage and selection operator (LASSO) regression was performed to identify variables that could predict Treg and Th17 cell induction by the OA isolates. R^2^ values for the model are depicted next to each cell population type, and important variables and their corresponding coefficients are below.

## Data Availability

Raw sequence data from RNA sequencing experiments are deposited in the NCBI Sequence Read Archive under BioProject accession number GSE262316.
