# Supplementary material for: Functional characterization of helminth-associated Clostridiales reveals covariates of Treg differentiation
Source: Microbiome. 2024 May 10;12:86. doi: 10.1186/s40168-024-01793-1 (PMC11084060; doi:10.1186/s40168-024-01793-1)
Supplement: Supplementary file 2 — Additional file 1: Figure S1. Enzymatic profile of the OA isolates in vivo. Representative images of α-chymotrypsin and β-glucuronidase enzymatic activity measured in fresh stool from mice monocolonized with the indicated OA isolates. GF, germ-free; SPF, Specific pathogen free. Figure S2. Bacterial burden and absolute numbers of Treg and Th17 cells induced by the OA isolates. (A) Colony forming units (CFUs) per mg of stool from mice colonized with indicated bacteria on the day cells were collected for analysis by flow cytometry. Each dot corresponds to one mouse. (B-D) Absolute numbers of Rorγt+ Helios− Foxp3+ Tregs (B), Rorγt+ Foxp3+ Tregs (C) and Rorγt+ Foxp3− Th17 cells (D) in the colon lamina propria of mice colonized with the indicated bacteria. SPF, Specific pathogen free; MF, Minimal flora mice; MF stool, GF mice gavaged with stool from Minimal flora mice; KH mix, Mice gavaged with consortium of 17 Clostridia strains from Atarashi et al., 2013; SFB, Segmented filamentous bacteria; GF, Germ-free. Each dot corresponds to one mouse. Each group was tested in at least 2 independent experiments consisting of 2–5 mice. Kruskal–Wallis with Dunn’s multiple comparisons test was used to compare between each group and GF in B-D. *p < 0.05, **p < 0.01, ***p < 0.001, ****p < 0.0001. Figure S3. Host transcriptional response to colonization by OA isolates. (A) PCA plot showing the overall transcriptional response (RNAseq) of cecal cells from GF mice and mice monocolonized with OA2, OA6 and OA8. Each dot corresponds to one mouse. (B-F) Heat maps of differentially expressed genes between pairs of groups. Genes with base mean > 50 and absolute value of logFC ≥ 2 are shown. (G-O) Expression of indicated genes between GF controls and monocolonized groups. Each dot represents one mouse. Bar graphs show mean ± SEM. Kruskal–Wallis with Dunn’s multiple comparisons test was used to compare between all groups. *p < 0.05. Figure S4. Genes correlated with Foxp3 expression. (A) Scatter pl [file 40168_2024_1793_MOESM1_ESM.pdf]

SUPPLEMENTAL FIGURES

Figure S1.

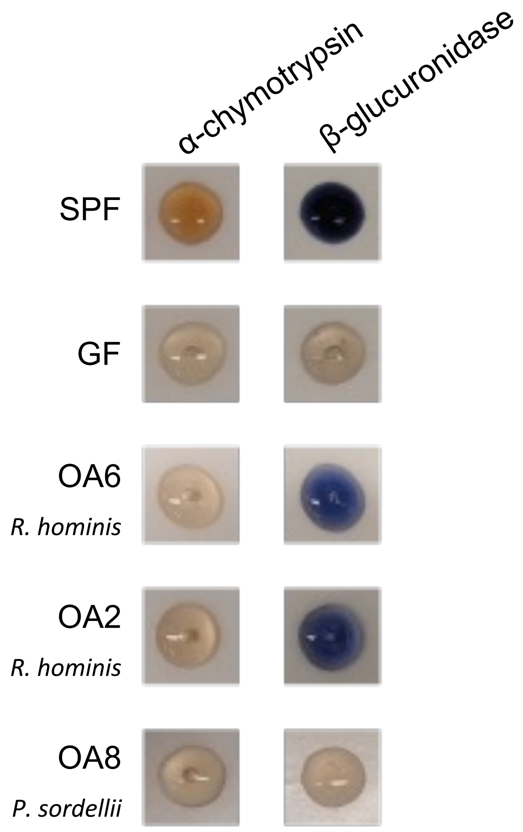

Figure S2.

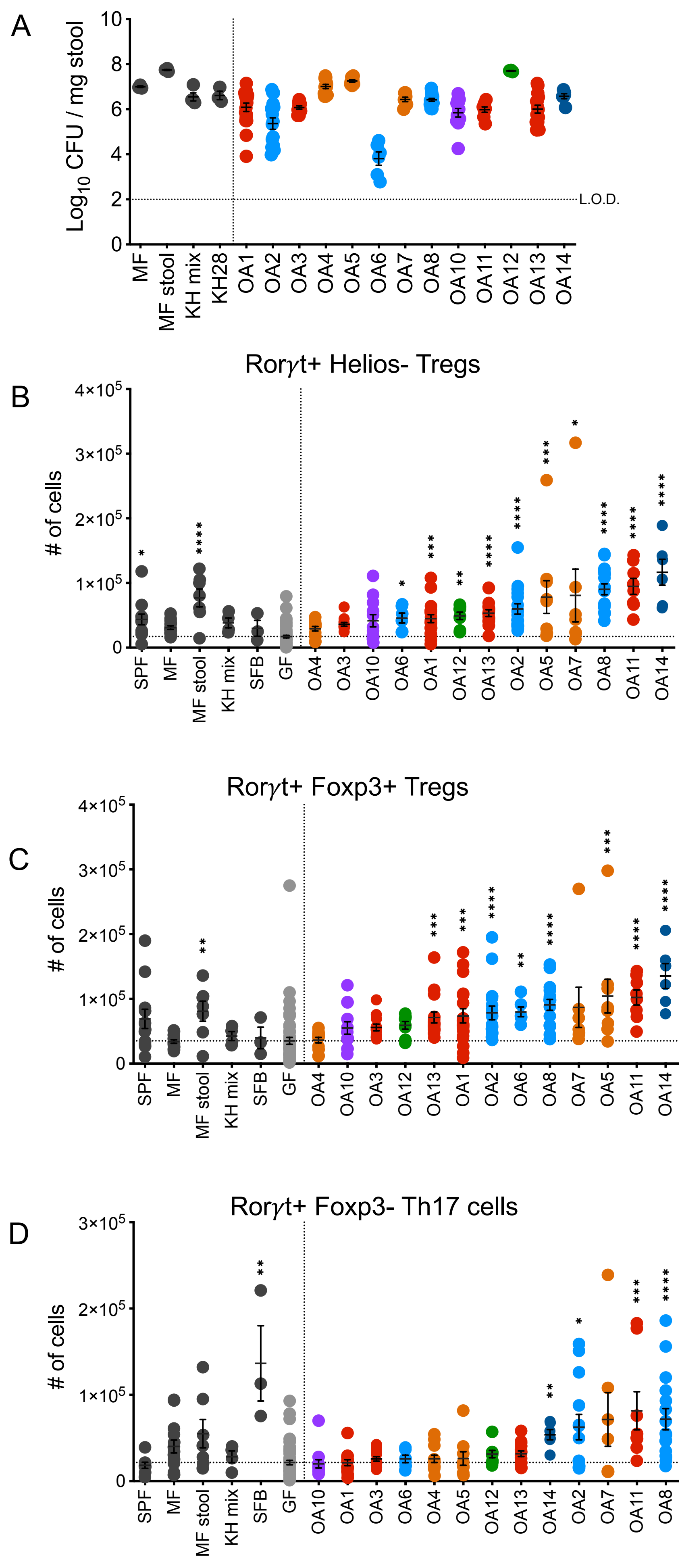
A
Log10 CFU / mg stool
10
8
6
4
2
0
L.O.D.
MF
MF stool
KH mix
KH28
OA1
OA2
OA3
OA4
OA5
OA6
OA7
OA8
OA10
OA11
OA12
OA13
OA14
B
Rorγt+ Helios- Tregs
# of cells
4×10^5
3×10^5
2×10^5
1×10^5
0
SPF
MF
MF stool
KH mix
SFB
GF
OA4
OA3
OA10
OA6
OA1
OA12
OA13
OA2
OA5
OA7
OA8
OA11
OA14
C
Rorγt+ Foxp3+ Tregs
# of cells
4×10^5
3×10^5
2×10^5
1×10^5
0
SPF
MF
MF stool
KH mix
SFB
GF
OA4
OA10
OA3
OA12
OA13
OA1
OA2
OA6
OA8
OA7
OA5
OA11
OA14
D
Rorγt+ Foxp3- Th17 cells
# of cells
3×10^5
2×10^5
1×10^5
0
SPF
MF
MF stool
KH mix
SFB
GF
OA10
OA1
OA3
OA6
OA4
OA5
OA12
OA13
OA14
OA2
OA7
OA11
OA8

Figure S3.

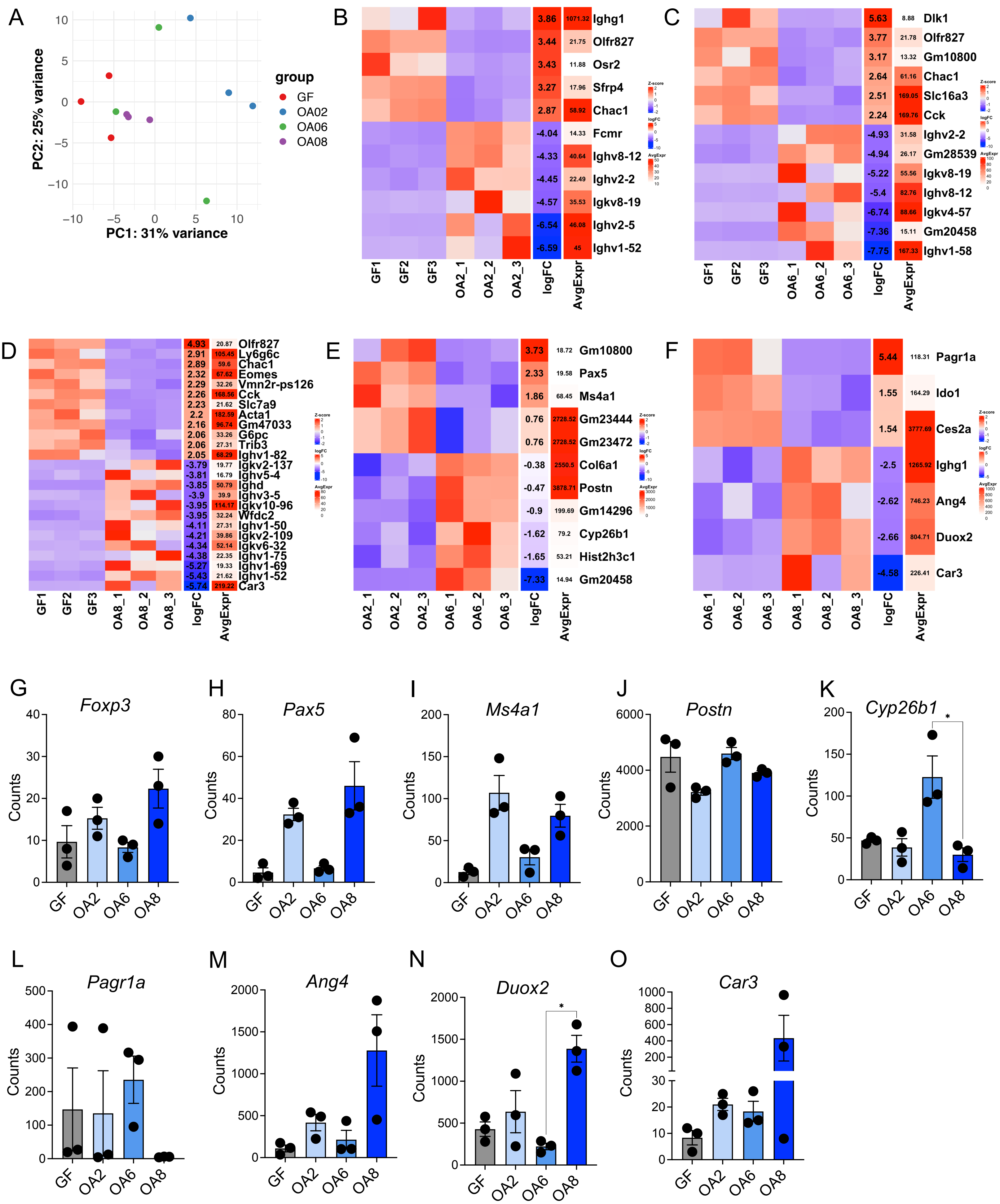

A
PC1: 31% variance
PC2: 25% variance
group
GF
OA02
OA06
OA08
B
Ighg1 3.86 1071.32
Olfr827 3.44 21.75
Osr2 3.43 11.88
Sfrp4 3.27 17.96
Chac1 2.87 58.92
Fcmr -4.04 14.33
Ighv8-12 -4.33 40.64
Ighv2-2 -4.45 22.49
Igkv8-19 -4.57 35.53
Ighv2-5 -6.54 46.08
Ighv1-52 -6.59 45
GF1 GF2 GF3 OA2_1 OA2_2 OA2_3 logFC AvgExpr
C
Dlk1 5.63 8.88
Olfr827 3.77 21.78
Gm10800 3.17 13.32
Chac1 2.64 61.16
Slc16a3 2.51 169.05
Cck 2.24 169.76
Ighv2-2 -4.93 31.58
Gm28539 -4.94 26.17
Igkv8-19 -5.22 55.56
Ighv8-12 -5.4 82.76
Igkv4-57 -6.74 88.66
Gm20458 -7.36 15.11
Ighv1-58 -7.75 167.33
GF1 GF2 GF3 OA6_1 OA6_2 OA6_3 logFC AvgExpr
D
Olfr827 4.93 20.87
Ly6g6c 2.91 105.45
Chac1 2.89 59.6
Eomes 2.32 67.62
Vmn2r-ps126 2.29 32.26
Cck 2.26 168.56
Slc7a9 2.23 21.62
Acta1 2.2 182.59
Gm47033 2.16 96.74
G6pc 2.06 33.26
Trib3 2.06 27.31
Ighv1-82 2.05 68.29
Igkv2-137 -3.79 19.77
Ighv5-4 -3.81 16.79
Ighd -3.85 50.79
Ighv3-5 -3.9 39.9
Igkv10-96 -3.95 114.17
Wfdc2 -3.95 32.24
Ighv1-50 -4.11 27.31
Igkv2-109 -4.21 39.86
Igkv6-32 -4.34 52.14
Ighv1-75 -4.38 22.35
Ighv1-69 -5.27 19.33
Ighv1-52 -5.43 21.62
Car3 -5.74 219.22
GF1 GF2 GF3 OA8_1 OA8_2 OA8_3 logFC AvgExpr
E
Gm10800 3.73 18.72
Pax5 2.33 19.58
Ms4a1 1.86 68.45
Gm23444 0.76 2728.52
Gm23472 0.76 2728.52
Col6a1 -0.38 2550.5
Postn -0.47 3878.71
Gm14296 -0.9 199.69
Cyp26b1 -1.62 79.2
Hist2h3c1 -1.65 53.21
Gm20458 -7.33 14.94
OA2_1 OA2_2 OA2_3 OA6_1 OA6_2 OA6_3 logFC AvgExpr
F
Pagr1a 5.44 118.31
Ido1 1.55 164.29
Ces2a 1.54 3777.69
Ighg1 -2.5 1265.92
Ang4 -2.62 746.23
Duox2 -2.66 804.71
Car3 -4.58 226.41
OA6_1 OA6_2 OA6_3 OA8_1 OA8_2 OA8_3 logFC AvgExpr
Z-score
logFC
AvgExpr
G Foxp3
H Pax5
I Ms4a1
J Postn
K Cyp26b1
L Pagr1a
M Ang4
N Duox2
O Car3
Counts
GF OA2 OA6 OA8
*

Figure S4.

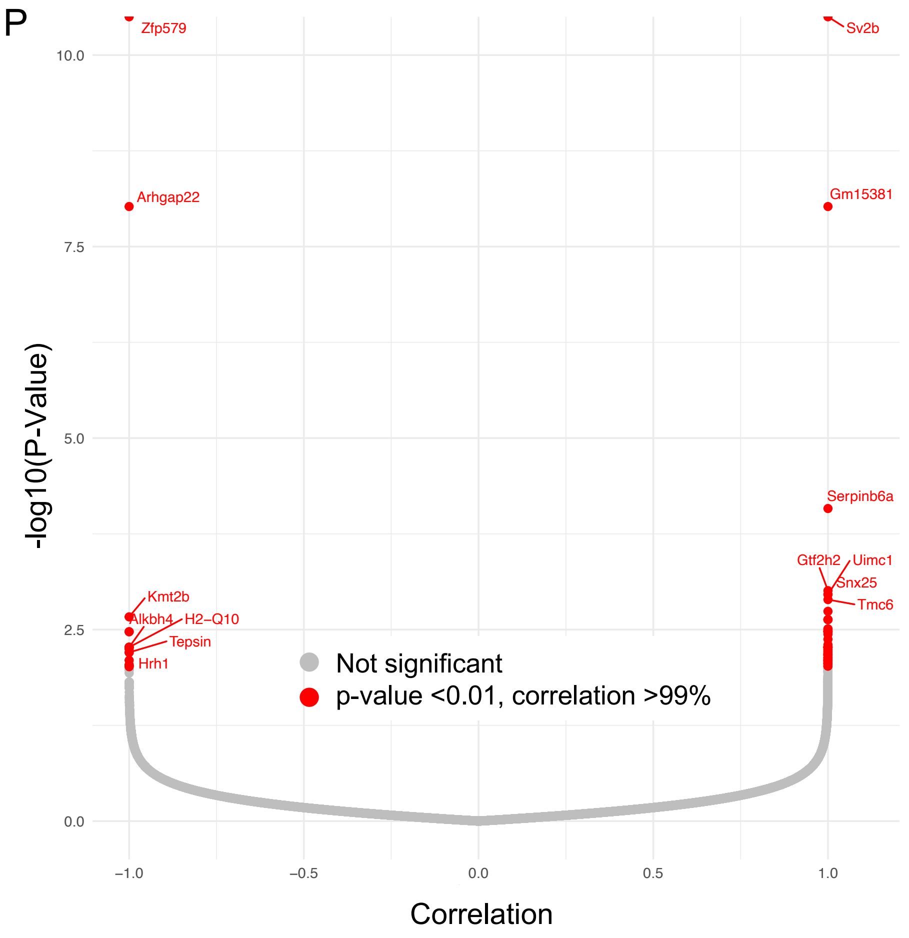
P
Zfp579
Sv2b
Arhgap22
Gm15381
Serpinb6a
Gtf2h2
Uimc1
Snx25
Tmc6
Kmt2b
Alkbh4
H2−Q10
Tepsin
Hrh1
Not significant
p-value <0.01, correlation >99%
10.0
7.5
5.0
2.5
0.0
-log10(P-Value)
−1.0
−0.5
0.0
0.5
1.0
Correlation

Figure S5.

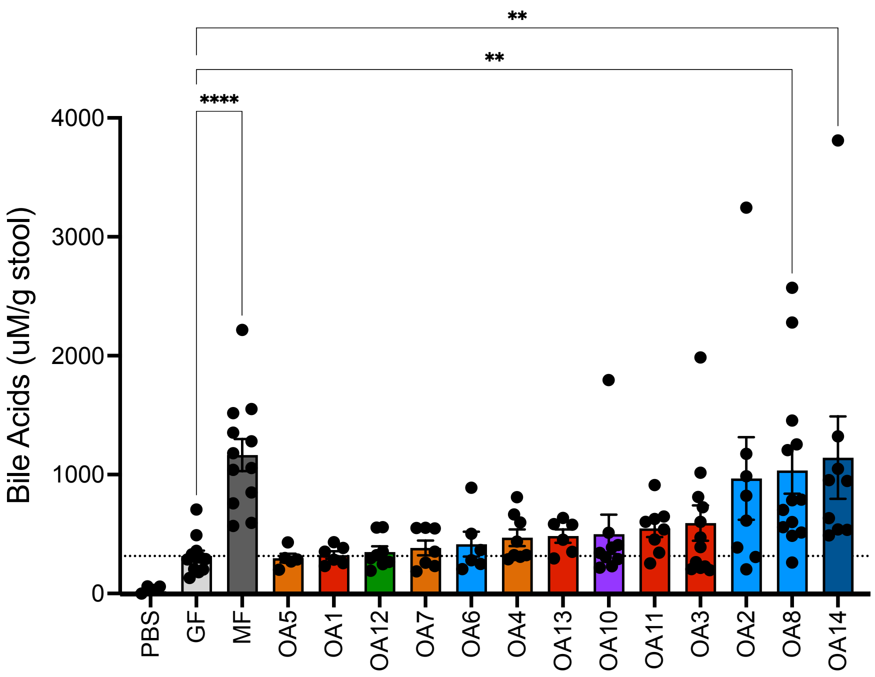
**
**
****
Bile Acids (uM/g stool)
4000
3000
2000
1000
0
PBS
GF
MF
OA5
OA1
OA12
OA7
OA6
OA4
OA13
OA10
OA11
OA3
OA2
OA8
OA14

Figure S6.

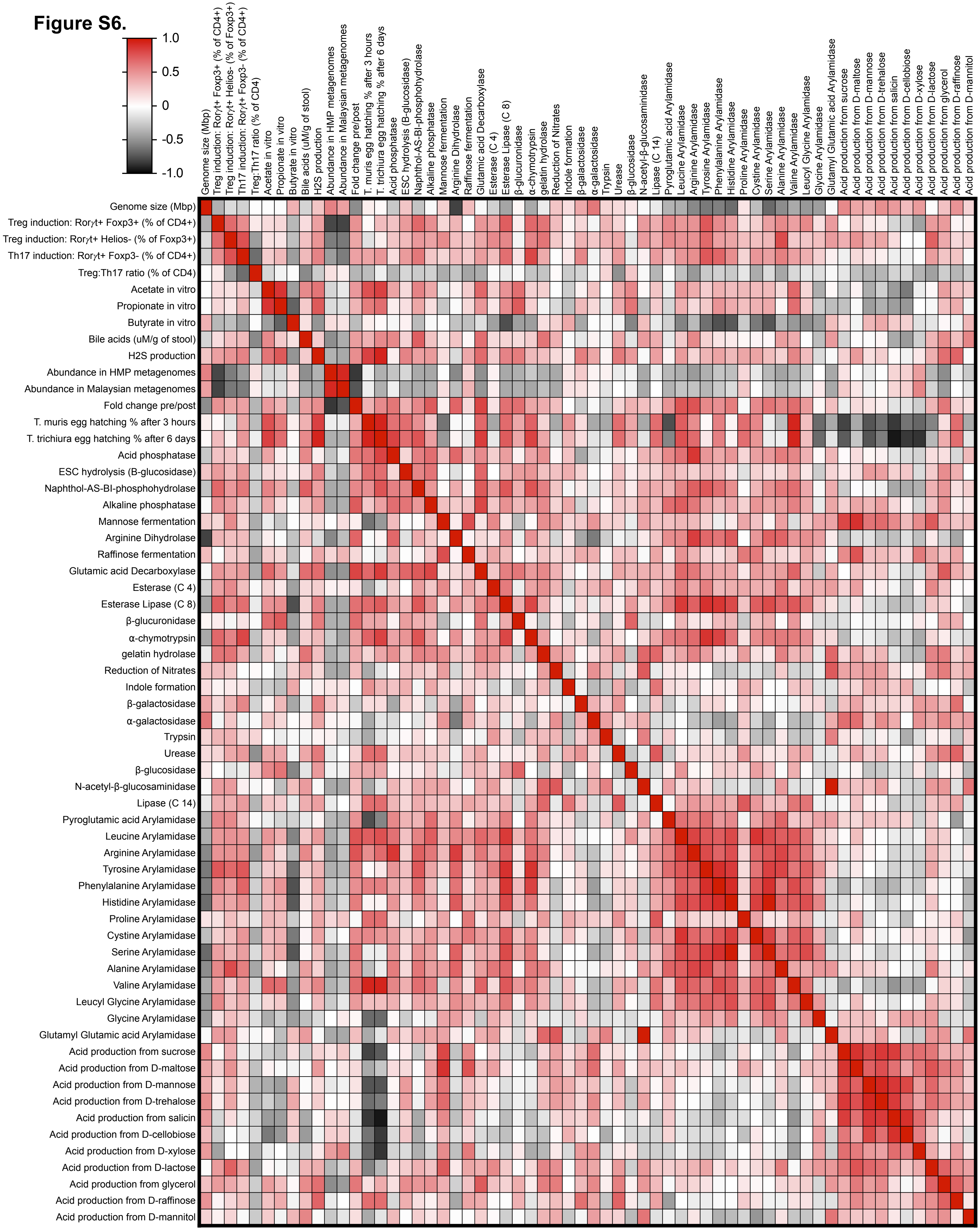
1.0
0.5
0
-0.5
-1.0
Genome size (Mbp)
Treg induction: Rorγt+ Foxp3+ (% of CD4+)
Treg induction: Rorγt+ Helios- (% of Foxp3+)
Th17 induction: Rorγt+ Foxp3- (% of CD4+)
Treg:Th17 ratio (% of CD4)
Acetate in vitro
Propionate in vitro
Butyrate in vitro
Bile acids (uM/g of stool)
H2S production
Abundance in HMP metagenomes
Abundance in Malaysian metagenomes
Fold change pre/post
T. muris egg hatching % after 3 hours
T. trichiura egg hatching % after 6 days
Acid phosphatase
ESC hydrolysis (B-glucosidase)
Naphthol-AS-BI-phosphohydrolase
Alkaline phosphatase
Mannose fermentation
Arginine Dihydrolase
Raffinose fermentation
Glutamic acid Decarboxylase
Esterase (C 4)
Esterase Lipase (C 8)
β-glucuronidase
α-chymotrypsin
gelatin hydrolase
Reduction of Nitrates
Indole formation
β-galactosidase
α-galactosidase
Trypsin
Urease
β-glucosidase
N-acetyl-β-glucosaminidase
Lipase (C 14)
Pyroglutamic acid Arylamidase
Leucine Arylamidase
Arginine Arylamidase
Tyrosine Arylamidase
Phenylalanine Arylamidase
Histidine Arylamidase
Proline Arylamidase
Cystine Arylamidase
Serine Arylamidase
Alanine Arylamidase
Valine Arylamidase
Leucyl Glycine Arylamidase
Glycine Arylamidase
Glutamyl Glutamic acid Arylamidase
Acid production from sucrose
Acid production from D-maltose
Acid production from D-mannose
Acid production from D-trehalose
Acid production from salicin
Acid production from D-cellobiose
Acid production from D-xylose
Acid production from D-lactose
Acid production from glycerol
Acid production from D-raffinose
Acid production from D-mannitol

Figure S7.

A

Explanatory variables used for dbRDA	ANOVA for each explanatory variable
Valine arylamidase	Pr(>F) = 0.003
α-chymotrypsin	Pr(>F) = 0.004
Alanine Arylamidase	Pr(>F) = 0.001
Tyrosine Arylamidase	Not significant
Esterase Lipase (C 8)	Pr(>F) = 0.028
Acid production from D-lactose	Pr(>F) = 0.002
Phenylalanine Arylamidase	Pr(>F) = 0.029
Naphthol-AS-BI-phosphohydrolase	Pr(>F) = 0.007
Gelatin hydrolase	Not significant
Acid production from salicin	Pr(>F) = 0.049
Acid production from D-trehalose	Pr(>F) = 0.045

B

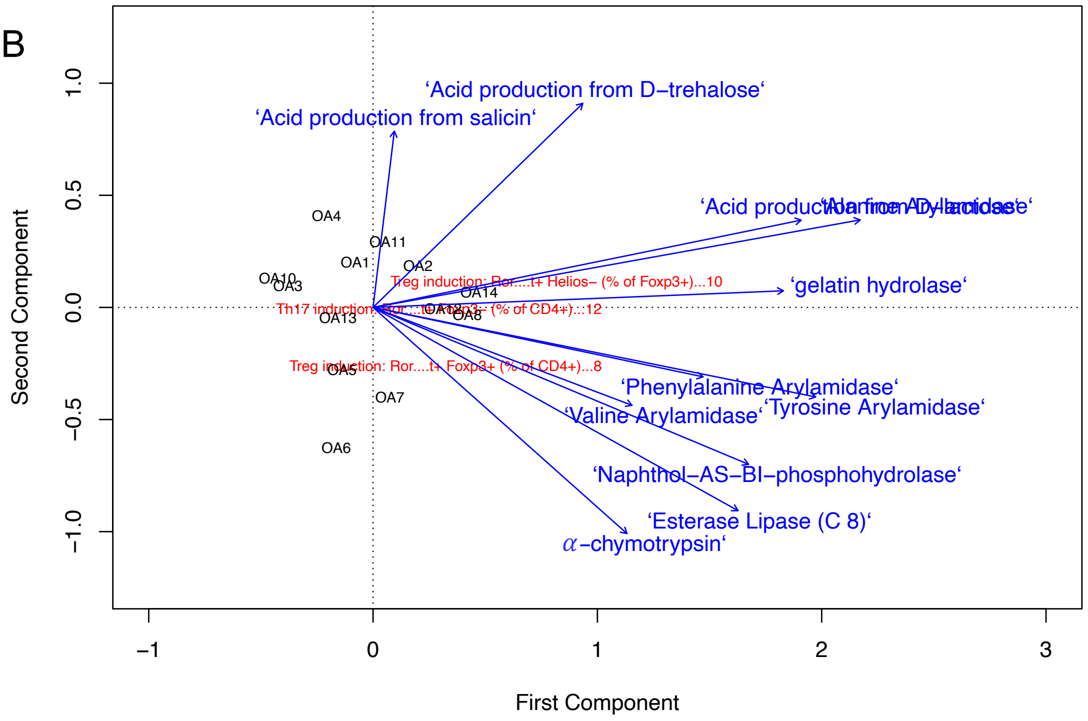

- **ANOVA for dbRDA model:** Pr(>F) = 0.009
- **ANOVA for dbRDA component 1:** Pr(>F) = 0.013 Explains 85% of the variance. Component 2 is not significant.
- **Proportion of constrained variance** > 0.99

C

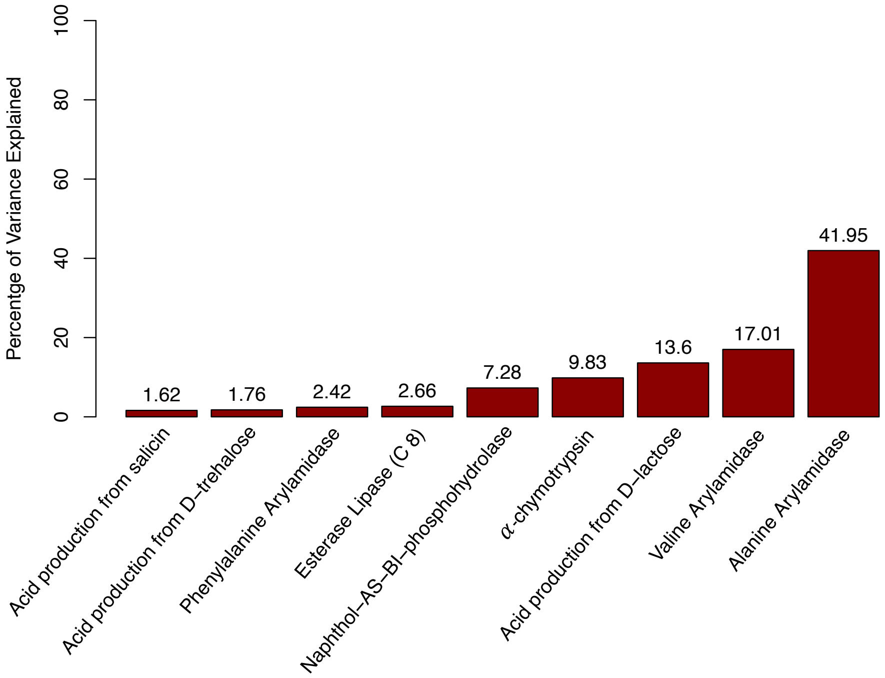

Figure S8.

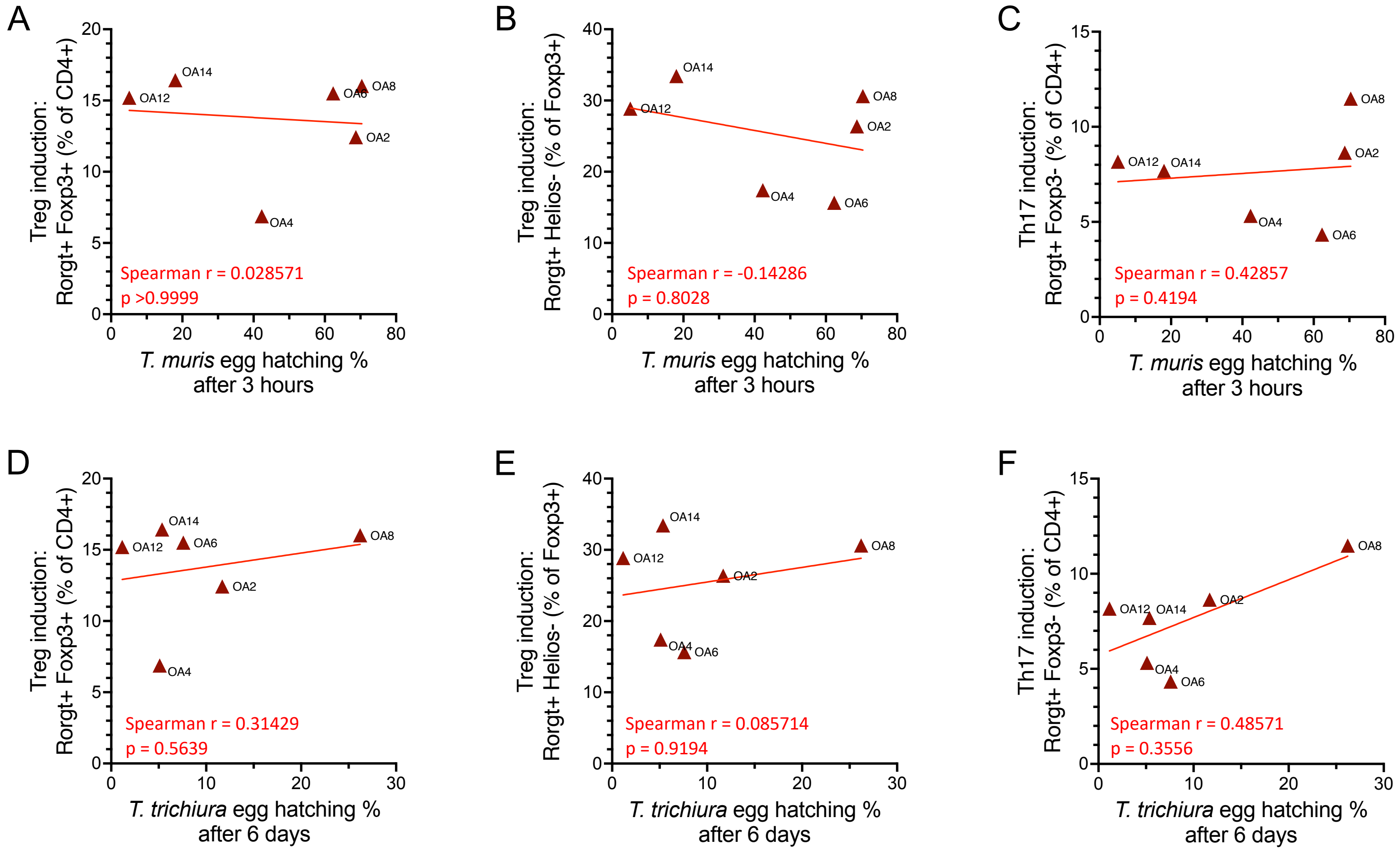
A
Treg induction:
Rorgt+ Foxp3+ (% of CD4+)
OA14
OA12
OA6
OA8
OA2
OA4
Spearman r = 0.028571
p >0.9999
T. muris egg hatching %
after 3 hours
B
Treg induction:
Rorgt+ Helios- (% of Foxp3+)
Spearman r = -0.14286
p = 0.8028
T. muris egg hatching %
after 3 hours
C
Th17 induction:
Rorgt+ Foxp3- (% of CD4+)
Spearman r = 0.42857
p = 0.4194
T. muris egg hatching %
after 3 hours
D
Treg induction:
Rorgt+ Foxp3+ (% of CD4+)
Spearman r = 0.31429
p = 0.5639
T. trichiura egg hatching %
after 6 days
E
Treg induction:
Rorgt+ Helios- (% of Foxp3+)
Spearman r = 0.085714
p = 0.9194
T. trichiura egg hatching %
after 6 days
F
Th17 induction:
Rorgt+ Foxp3- (% of CD4+)
Spearman r = 0.48571
p = 0.3556
T. trichiura egg hatching %
after 6 days
