# Supplementary material for: Functional characterization of helminth-associated Clostridiales reveals covariates of Treg differentiation
Source: Microbiome. 2024 May 10;12:86. doi: 10.1186/s40168-024-01793-1 (PMC11084060; doi:10.1186/s40168-024-01793-1)
Supplement: Supplementary file 3 — Additional file 2: Table S1. Fecal bacterial loads of mice in the study after ~ 8 weeks of inoculation. Germ-free mice (GF), mice colonized with OA02, OA06, and OA08 strains, and standard laboratory mice with a full microbiota (C57BL/6). Bacterial load was measured in a flow cytometer by counting fecal bacteria stained with a fluorescent DNA marker. Bacterial load is almost two orders of magnitude larger in monocolonized mice than in GF mice, and three orders of magnitude larger in standard mice than in germ-free mice. [file 40168_2024_1793_MOESM2_ESM.docx]

**Table S1.** Fecal bacterial loads of mice in the study after ~8 weeks of inoculation. Germ-free mice (GF), mice colonized with OA02, OA06, and OA08 strains, and standard laboratory mice with a full microbiota (C57BL/6). Bacterial load was measured in a flow cytometer by counting fecal bacteria stained with a fluorescent DNA marker. Bacterial load is almost two orders of magnitude larger in monocolonized mice than in GF mice, and three orders of magnitude larger in standard mice than in germ-free mice.

| Colonization status of mouse | Fecal Bacterial Load (10^6 cells/g) |
| --- | --- |
| GF | 0.025 |
| GF | 0.016 |
| GF | 0.013 |
| OA02 | 0.759 |
| OA02 | 0.418 |
| OA02 | 0.543 |
| OA06 | 0.509 |
| OA06 | 0.401 |
| OA06 | 0.653 |
| OA08 | 0.699 |
| OA08 | 0.56 |
| OA08 | 0.684 |
| C57BL/6 | 57.33 |
| C57BL/6 | 51.42 |
