# Supplementary material for: Functional characterization of helminth-associated Clostridiales reveals covariates of Treg differentiation
Source: Microbiome. 2024 May 10;12:86. doi: 10.1186/s40168-024-01793-1 (PMC11084060; doi:10.1186/s40168-024-01793-1)
Supplement: Supplementary file 4 — Additional file 3: Table S2. Least absolute shrinkage and selection operator (LASSO) regression was performed to identify variables that could predict Treg and Th17 cell induction by the OA isolates. R2 values for the model are depicted next to each cell population type, and important variables and their corresponding coefficients are below. [file 40168_2024_1793_MOESM3_ESM.docx]

**Table S2.** Least absolute shrinkage and selection operator (LASSO) regression was performed to identify variables that could predict Treg and Th17 cell induction by the OA isolates. R^2^ values for the model are depicted next to each cell population type, and important variables and their corresponding coefficients are below.

| **Treg induction: Ror𝛾t+ Foxp3+ (% of CD4+),** R^2^ > 0.3 | |
| --- | --- |
| Variable | Coefficient |
| Esterase Lipase (C 8) | 1.73E-02 |
| **Treg induction: Ror𝛾t+ Helios- (% of Foxp3+),** R^2^ > 0.99 | |
| Variable | Coefficient |
| Alanine Arylamidase | 3.44E-01 |
| Alpha-Chymotrypsin | 6.94E-02 |
| Beta-galactosidase | 4.02E-02 |
| Alpha-Arabinosidase | -3.88E-02 |
| Gelatin Hydrolase | 3.27E-02 |
| Acid production from L-rhamnose | 2.97E-02 |
| Acid production from D-trehalose | 2.92E-02 |
| Acid production from D-maltose | 2.68E-02 |
| Acid production from D-glucose | 1.64E-02 |
| Pyroglutamic Acid Arylamidase | 1.33E-02 |
| **Th17 cell induction: Ror𝛾t+ Foxp3- (% of CD4+),** R^2^ > 0.97 | |
| Variable | Coefficient |
| Beta-Galactosidase | 1.24E-01 |
| Beta-Galactosidase 6 Phosphate | -5.88E-02 |
| Arginine Arylamidase | 4.77E-02 |
| Alpha-Chymotrypsin | 3.19E-02 |
| Alanine Arylamidase | 2.19E-02 |
| Cysteine Arylamidase | 1.12E-02 |
| Acid Production from D-trehalose | 1.10E-02 |
| Acid Production from D-sorbitol | 4.18E-03 |
| ESC Hydrolysis (B-glucosidase) | 3.94E-04 |
